# Surface Charge Overrides Protein Corona Formation in Determining the Cytotoxicity, Cellular Uptake, and Biodistribution of Silver Nanoparticles

**DOI:** 10.1021/acsabm.5c00392

**Published:** 2025-05-21

**Authors:** Marianna Barbalinardo, Francesca Chiarini, Gabriella Teti, Francesca Paganelli, Elisa Mercadelli, Andrea Bartoletti, Andrea Migliori, Manuela Piazzi, Jessika Bertacchini, Paola Sena, Alessandra Sanson, Mirella Falconi, Carla Palumbo, Massimiliano Cavallini, Denis Gentili

**Affiliations:** † Consiglio Nazionale delle Ricerche, Istituto per lo Studio dei Materiali Nanostrutturati (CNR-ISMN), via P. Gobetti 101, 40129 Bologna, Italy; ‡ Department of Biomedical, Metabolic and Neural Sciences, Section of Human Morphology, 9306 University of Modena and Reggio Emilia, via del Pozzo 71, 41124 Modena, Italy; § Department of Biomedical and Neuromotor Sciences, University of Bologna, via Irnerio 48, 40126 Bologna, Italy; ∥ Consiglio Nazionale delle Ricerche, Istituto di Scienza, Tecnologia e Sostenibilità per lo Sviluppo dei Materiali Ceramici (ISSMC), via Granarolo 64, 48018 Faenza, Italy; ⊥ Consiglio Nazionale delle Ricerche, Istituto di Genetica Molecolare (CNR-IGM), via Di Barbiano 1/10, 40136 Bologna, Italy; # Department of Surgery, Medicine Dentistry and Morphological Sciences with Interest in Transplant, University of Modena and Reggio Emilia, via del Pozzo 71, 41124 Modena, Italy; ∇ Department of Medical and Surgical Sciences, University of Bologna, via Irnerio 48, 40126 Bologna, Italy; $ IRCCS Istituto Ortopedico Rizzoli, 40136 Bologna, Italy

**Keywords:** Nanoparticles, Protein corona, Surface charge, Cell uptake, Cytotoxicity, Biodistribution

## Abstract

Silver nanoparticles (AgNPs) hold great promise in biomedical applications due to their unique properties and potential for specific tissue targeting. However, the clinical translation of nanoparticle-based therapeutics remains challenging, primarily due to an incomplete understanding of how nanoparticle properties influence interactions at the nano–bio interface, as well as the role of surface-adsorbed proteins (i.e., protein corona) in modulating nanoparticle–cell interactions. This study demonstrates that the surface charge has a greater influence than protein corona formation in determining the cytotoxicity, cellular uptake, and biodistribution of AgNPs. Using negatively and positively charged AgNPs, we show that while protein corona formation is essential for ensuring nanoparticle availability for cellular interactions, the adsorption of biomolecules is nonspecific and independent of surface charge. Conversely, the surface charge significantly influences the interactions of AgNPs with cells. Positively charged nanoparticles exhibit enhanced cellular uptake, preferential accumulation in lysosomes, and pronounced mitochondrial damage compared to their negatively charged counterparts, resulting in greater cytotoxic effects. This effect is particularly evident in human breast cancer cells, where negatively charged nanoparticles show minimal uptake and cytotoxicity. These findings demonstrate that surface charge is the primary factor governing nanoparticle–cell interactions rather than protein corona formation. Nonetheless, the protein corona plays a critical role in stabilizing nanoparticles in physiological environments.

## Graphical abstract

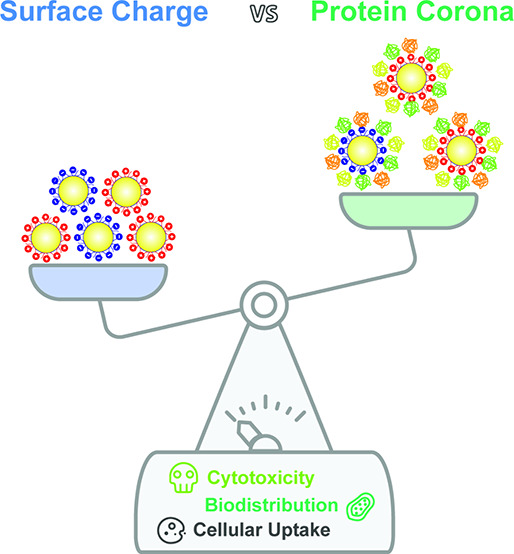

## 1 Introduction

Nanoparticles (NPs) are increasingly studied because their engineering, combined with their unique properties, opens up a myriad of possibilities in various biomedical applications.^1,2^ As recently demonstrated by the development of mRNA-based vaccines against COVID-19, NPs are no longer just a promise but real tools on which we can rely on.^3,4^ However, while vaccines need to stimulate the immune cells, therapeutics must be delivered to specific tissues, and to date, fundamental challenges remain regarding the targeting of NPs.^3^ So far, despite many successful preclinical trials, only a few passively targeted nanocarriers are approved for clinical use, and none of the actively targeted nanoparticles have been approved.^5^ Many challenges still need to be addressed to make the clinical translation of nanoparticle-based therapeutics more efficient, including reaching a comprehensive understanding of nano–bio interactions.^6^ It is well-acknowledged that the biological activity of NPs is intricately linked to their physicochemical properties. Yet, despite this understanding, accurately predicting the impact of NPs on biological systems remains a challenge.^7^ One of the factors complicating the understanding of interactions at the nano–bio interface is the formation of the protein corona, which is a dynamic layer of biomolecules that adsorbs onto the surface of nanoparticles when exposed to physiological environments. This process can drastically change the properties of nanoparticles and, in turn, their interactions with cells, significantly affecting their trafficking into tissues, i.e., recognition by the immune system, blood circulation time, biodistribution, and endocytosis.^8–19^ Among all physicochemical properties, such as size, shape, and aggregation state, the surface charge of NPs has been reported as a key characteristic in determining their interaction with biological entities.^20–29^ However, the interplay between nanoparticle surface charge and protein corona formation remains poorly understood, prompting questions about how the former influences the latter and which of the two predominantly governs the interactions between nanoparticles and cells.^21^ Among nanomaterials, silver NPs (AgNPs) have emerged as a versatile and promising class of nanomaterial, finding applications across a broad spectrum of fields including medicine and environmental science. Their exceptional antimicrobial properties and potential for use in drug delivery systems, imaging technologies, and therapeutics underscore their significance. However, the safe and effective application of AgNPs in the biomedical context necessitates a comprehensive understanding of their interactions with biological systems.^30–36^ We recently reported that the adsorption of proteins onto the surface of negatively charged citrate-capped AgNPs, followed by the formation of a protein corona, mediates their cellular uptake and cytotoxicity. In contrast, uncharged AgNPs capped with an oligo­(ethylene glycol)-terminated alkanethiol are more resistant to protein adsorption on their surface and are nontoxic.^37–39^ However, the contribution of surface charge, protein corona formation, and their interplay to the interactions of AgNPs with cells remains largely not well understood.

In this study, we investigate the relationship between surface charge and protein corona formation and their effects on cytotoxicity, cellular uptake, and biodistribution as well as the morphological impact of AgNPs in human cancer cells and mouse fibroblasts. By employing AgNPs with distinct surface charges, we show that the cytotoxic activity, biodistribution, and cellular uptake of AgNPs are charge-dependent, whereas the adsorption of proteins from the cellular medium onto the surface of the nanoparticles, leading to the formation of a protein corona, is a process independent of surface charge. Our results reveal that while protein corona formation is essential to stabilize nanoparticles in physiological environments and ensure their availability for cellular uptake, it is the surface charge that predominantly dictates the intracellular fate and cytotoxic effects of AgNPs. These findings underscore the crucial role of surface charge in shaping interactions at the nano–bio interface, with significant implications for the design and biomedical application of AgNPs, particularly regarding their mechanism of action and cytotoxicity.

## 2 Results and Discussion

### 2.1 Physicochemical Characterization

Citrate-coated silver nanoparticles (AgNPs-cit) with an average diameter of 20 ± 1 nm, as determined through transmission electron microscopy (TEM) analysis, were synthesized according to a previously reported procedure^40,41^ and subsequently modified using layer-by-layer deposition techniques (see the Experimental Section), resulting in a diverse array of surface charges. Specifically, as shown in Figure 1a,b, we prepared negatively charged citrate- and poly­(sodium 4-styrene­sulfonate) (PSS)-coated AgNPs, as well as positively charged poly­(allylamine hydrochloride) (PAH)- and poly­(diallyl­dimethyl­ammonium chloride) (PDDA)-coated AgNPs. Polyelectrolyte (PE) coating conditions were adapted from previously reported procedures and optimized to achieve stable NPs with a robust and evenly distributed surface charge while keeping any possible size variation to a minimum.^42,43^ The resulting polyelectrolyte layer is a few nanometers, as evidenced by TEM images (Figure 1c); in fact, the average hydrodynamic size of the AgNPs-cit, as determined by dynamic light scattering (DLS) measurements, was slightly increased after coating with PEs, while the surface charge varies according to the coating exposed on the surface (see Figure 1d). Furthermore, we noted the absence of broadening and shape changes in the profiles of the absorption spectra (Figure 1e), thus revealing the absence of the aggregation of AgNPs induced by the PE coating.

**Figure 1:**
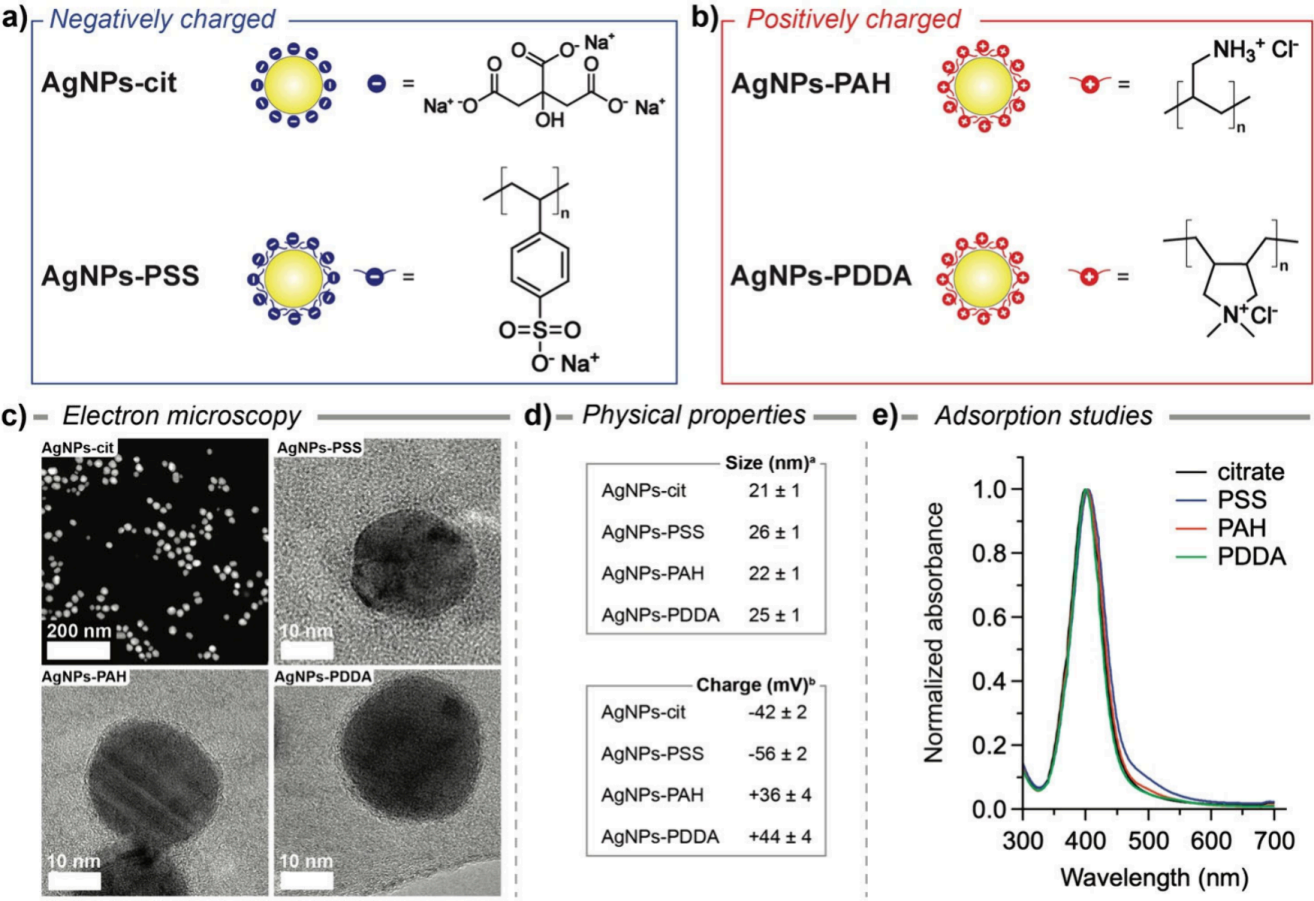
Schematic representation of (a) negatively (AgNPs-cit and AgNPs-PSS) and (b) positively (AgNPs-PAH and AgNPs-PDDA) charged AgNPs. (c) STEM image of (top left) AgNPs-cit, and TEM images of (top right) AgNPs-PSS, (bottom left) AgNPs-PAH, and (bottom right) AgNPs-PDDA stained with phosphotungstic acid (see the Experimental Section). Scale bar: 200 nm STEM and 10 nm TEM. (d) Physical properties of (top) size (^a^hydrodynamic diameter) and (bottom) surface charge (^b^ *ζ*-potential) as a function of surface coating. (e) UV–vis absorption spectra of AgNPs in deionized water as a function of surface coating.

### 2.2 Cytotoxicity of Nanoparticles

We evaluated the cytotoxicity of AgNPs on a fibroblast cell line (NIH-3T3) and human cancer cell lines (MCF7, breast cancer cell line; Caco2, colon cancer cell line). The cell viability was assessed by measuring mitochondrial activity using a 3-(4,5-dimethyl-2-thiazolyl)-2,5-diphenyl­tetra­zolium bromide (MTT) assay after 24 and 48 h exposures to 20 μg/mL AgNPs with different surface charges. As shown in Figure 2a, the cell viability assay unveiled a significant time-dependent decrease in the mitochondrial function in NIH-3T3 and Caco2 cells following exposure to AgNPs. This trend was observed both with negatively charged nanoparticles, AgNPs-cit and AgNPs-PSS, and positively charged ones, AgNPs-PAH and AgNPs-PDDA. In the case of MCF7 cells, it was observed that even after 48 h of exposure to negatively charged NPs, cell viability did not seem to be significantly affected. Conversely, the treatment with positively charged NPs resulted in a significant decrease in cell viability after only 24 h of exposure, and this effect was even more pronounced after 48 h. In fact, as revealed by the Tukey test analysis in Figure 2a, the cellular activity in the presence of positively charged NPs differs significantly from the cellular activity in the presence of negatively charged nanoparticles. Note that the supernatant obtained after centrifugation of all AgNP solutions did not exhibit any significant cytotoxicity in any of the three cell lines, even after 48 h of incubation (Figure S1). This confirms that the observed cytotoxic effects can be attributed to the nanoparticles themselves rather than to any potentially residual free polymer or ligand present in the solution.

**Figure 2:**
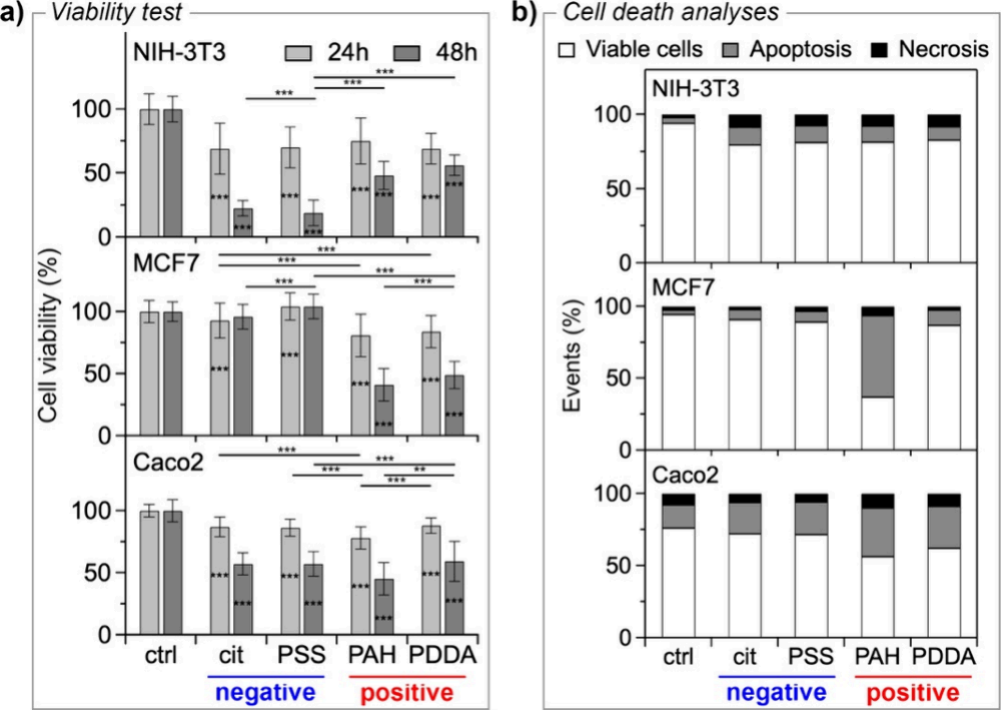
(a) Cell viability of NIH-3T3, MCF7, and Caco2 cells treated for 24 and 48 h with AgNPs (20 μg/mL) as a function of surface coating (AgNPs-cit and AgNPs-PSS negatively charged; AgNPs-PAH and AgNPs-PDDA positively charged). Data represent the mean ± SD and are plotted as a percentage in reference to control samples (ctrl). At least seven independent experiments, each with 10 biological replicates, were carried out, and statistical analyses were performed using ANOVA followed by Tukey’s test. **p < 0.01 and ***p < 0.001 denote significant differences with respect to the control. (b) Flow cytometric analysis of Annexin V and PI staining of NIH-3T3, MCF7, and Caco2 cells treated for 24 (NIH-3T3 and Caco2) or 48 (MCF7) h with AgNPs (20 μg/mL) as a function of surface coating (AgNPs-cit and AgNPs-PSS negatively charged; AgNPs-PAH and AgNPs-PDDA positively charged). At least three independent experiments, each with three biological replicates, were carried out.

To gain further insights into these results, which demonstrated the surface charge-dependent toxicity of AgNPs, we then analyzed cell death using flow cytometry. Cells were double-stained with Annexin V-FITC and propidium iodide (PI) after treatment with AgNPs for the indicated times. As shown in Figure 2b, NIH-3T3 cells treated with AgNPs exhibited increased levels of apoptosis and necrosis compared to the control, already visible after 24 h. A similar trend was seen for apoptosis in Caco2 cells. In both cell types, this increase is independent of the surface charge of the NPs, with no significant differences based on the chemical nature of the coating. Conversely, MCF7 cells did not show a significant increase in cell death, and only a slight increase in apoptotic cells was observed upon treatment with negatively charged NPs. On the other hand, a considerable increase in apoptosis was observed with positively charged NPs compared to the control after 48 h of treatment. This increase in apoptosis, accompanied by a modest increase in necrosis, is more pronounced in cells treated with AgNPs-PAH than in cells treated with AgNPs-PDDA. However, this difference may be due to the higher uptake of the former NPs compared to the latter (see below). Therefore, according to our results, the surface charge plays a key role in the cytotoxic activity of NPs toward MCF7 cells, particularly in the apoptosis process, whereas it does not have the same effect on NIH-3T3 and Caco2 cells.

### 2.3 Uptake of Nanoparticles

The cellular uptake was assessed by inductively coupled plasma optical emission spectroscopy (ICP-OES) analyses. For this purpose, cells were incubated for 16 h with 20 μg/mL AgNPs and then extensively washed. A shorter incubation time, compared with that used for cell viability studies, was chosen to ensure the uptake of the NPs while avoiding cell detachment due to their cytotoxic activity. In fact, the cell number for the samples exposed to nanoparticles was found to be similar to that of cells treated with the vehicle solution (see the Experimental Section). Figure 3a shows the ratios of silver per cell, representing AgNPs that were either firmly attached to the cell membrane or internalized within the cells. The uptake of positively charged AgNPs, whether coated with PAH or PDDA, is significantly higher than that of negatively charged ones. This trend is observed across all three cell lines tested and demonstrates that the surface charge significantly influences the interaction of AgNPs with cells. This result is in line with results reported in studies on other nanoparticles, such as gold NPs, polystyrene NPs, poly­(ethylene glycol)-d,l-polylactide NPs, cerium oxide NPs, silica NPs, carbon dots, and quantum dots.^22–29,44–46^ The pronounced tendency of positively charged AgNPs to engage with cells, irrespective of the cell line, implies that electrostatic interactions play a pivotal role in the interaction between NPs and the cell membrane.^14,26,46,47^ It is interesting to note that while PSS-coated AgNPs are not internalized by MCF7 cells within the detectable limits of the employed technique, there is a slight uptake of citrate-coated AgNPs. However, as seen previously, this uptake is not sufficient to induce a significant cytotoxic effect.

**Figure 3:**
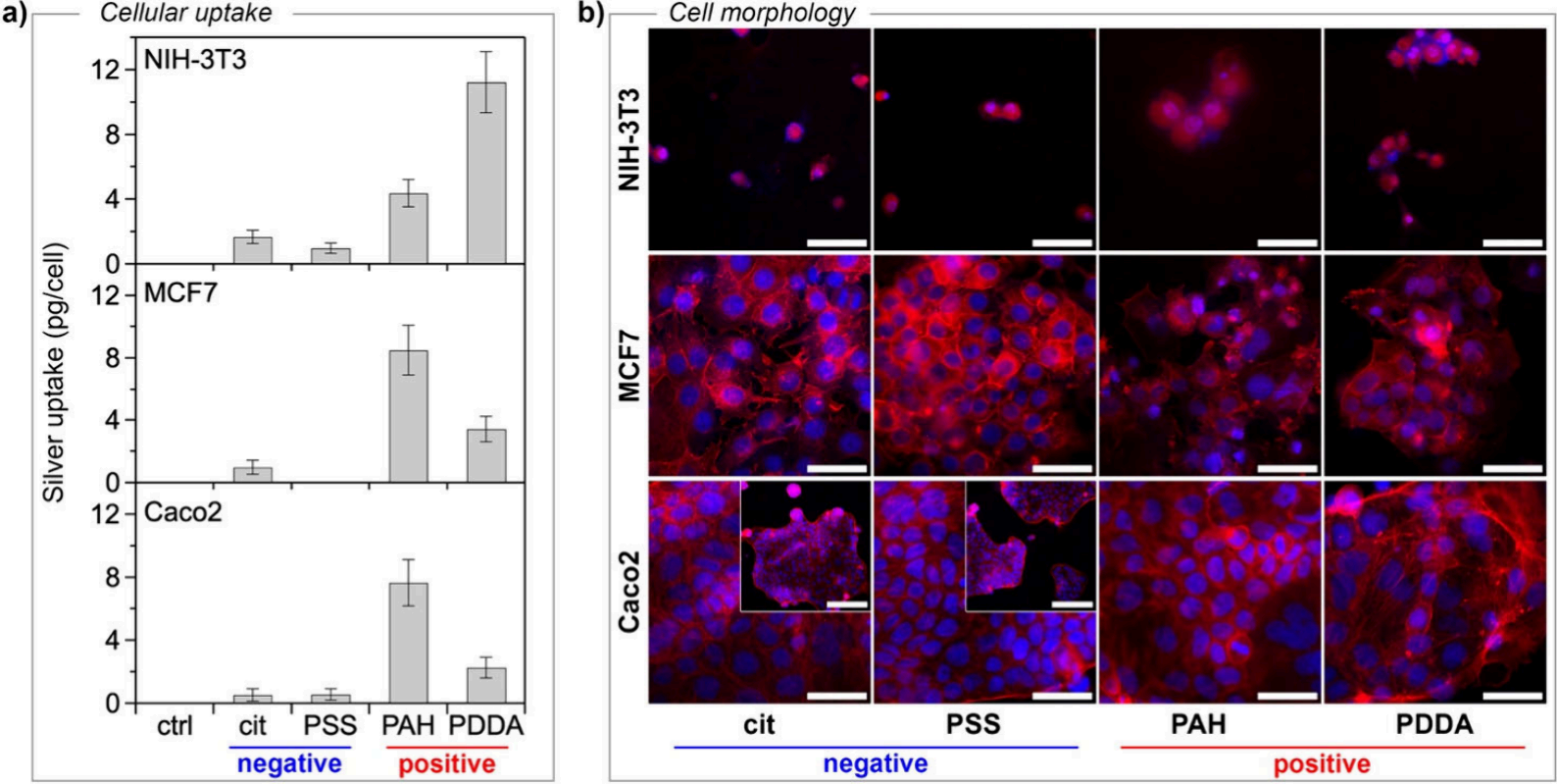
(a) Cellular uptake of silver by NIH-3T3, MCF7, and Caco2 cells after 16 h of exposure to 20 μg/mL AgNPs as a function of surface coating. Data are presented as mean ± SD. At least three independent experiments, each with three biological replicates, were carried out. (b) Fluorescence micrographs of NIH-3T3, MCF7, and Caco2 cells labeled specifically for actin (red) and the nucleus (blue) and treated with AgNPs (20 μg/mL) as a function of surface coating after 48 h of incubation (scale bar: 50 μm). Scale bar in insets: 200 μm.

### 2.4 Effect on Cell Morphology

We have investigated the structure of cells through fluorescence labeling of the nucleus (blue) and actin filaments (red), which are pivotal in regulating cell functions such as proliferation, adhesion, differentiation, and internal architecture. As shown in Figure 3b (first row), both negative and positive nanoparticles drastically deteriorate actin filaments of NIH-3T3 cells and compromise their morphology. A significant decrease in cell density and a reduction in cytoskeleton size are also observed, indicating reduced cell adhesion to the substrate and impaired replicative ability compared to cells treated with the control (Figure S2a). Consistent with the viability results, Caco2 cells exhibited similar behavior (Figure 3b, third row). There is a clear overall impact on cell morphology and a significant decrease in cell density compared to the control (Figure S2c), regardless of the type of coating or surface charge. On the contrary, in MCF7 cells (Figure 3b, second row), the impact of nanoparticles varied significantly depending on their surface charge. Positively charged AgNPs significantly influenced cell shape and cytoskeletal dynamics, whereas negatively charged AgNPs had no discernible effect compared to control-treated cells (Figure S2b).

### 2.5 Biodistribution of Nanoparticles

Ultrastructural analysis by TEM was performed to evaluate the biodistribution of nanoparticles in NIH-3T3, MCF7, and Caco2 cells after 6 and 24 h of incubation with 20 μg/mL AgNPs and the consequent intracytoplasmic morphological changes due to this treatment. Untreated NIH-3T3 cells showed a spindle-like morphology and well-preserved cellular organelles with no abnormalities (Figure S3a,b). On the other hand, NIH-3T3 fibroblasts treated for 6 h with negatively charged citrate- and PSS-coated AgNPs exhibited a distinctive polygonal and spindle-like morphology with no evident damage apparent in the cellular structure (Figure 4a,c). However, closer inspection at higher magnification revealed the presence of AgNPs in the cytoplasm (black arrows in Figure 4b, inset) and several damaged mitochondria (Figure 4b,d). These mitochondria exhibited signs of distress, such as swelling and disruption of internal cristae, highlighting an impact at the subcellular level. After 24 h of exposure, clusters of negatively charged AgNPs were found in the cytoplasm (black arrow in Figure S4b, inset). Although the cells still exhibited preserved morphology, the damage to the mitochondria became even more evident (Figure S4a–d), and the chromatin revealed small areas of condensation (Figure S4c), indicating early events of apoptosis. Even after 6 h of exposure to positively charged PAH- and PDDA-coated AgNPs, NIH-3T3 fibroblasts maintained a preserved fibroblast-like shape (Figure 4e,g). Nevertheless, the NPs were found to be distributed inside lysosomes (black arrows in Figure 4f,h, inset), and morphological changes were already noticeable in both the nucleus and the cytoplasm. Small areas of condensed chromatin along with several empty and dilated mitochondria were observed in cells treated with PAH-coated AgNPs (Figure 4e). Irregular mitochondria with extracted internal cristae and several myelin figures were detected in the cytoplasm of cells treated with PDDA-coated AgNPs, suggesting early mitochondrial cytotoxic effects followed by degrading events (Figure 4h). After 24 h of exposure, NIH-3T3 cells treated with positively charged AgNPs exhibited a high level of necrosis (Figure S4e,g), and clusters of nanoparticles were observed inside the cytoplasm (black arrows in Figure S 4f–h). In the case of cells exposed to PAH-coated AgNPs, the nucleus remained detectable, but the chromatin was greatly extracted, and the cytoplasm suffered extensive damage. Additionally, no distinguishable cellular organelles were observed (Figure S4e,f). Similarly, exposure to PDDA-coated AgNPs led to nuclei degradation and aggregated chromatin, resembling the final stages of apoptosis. The cytoplasm also exhibited significant degradation, making it challenging to detect any cellular organelles (Figure S4g).

**Figure 4:**
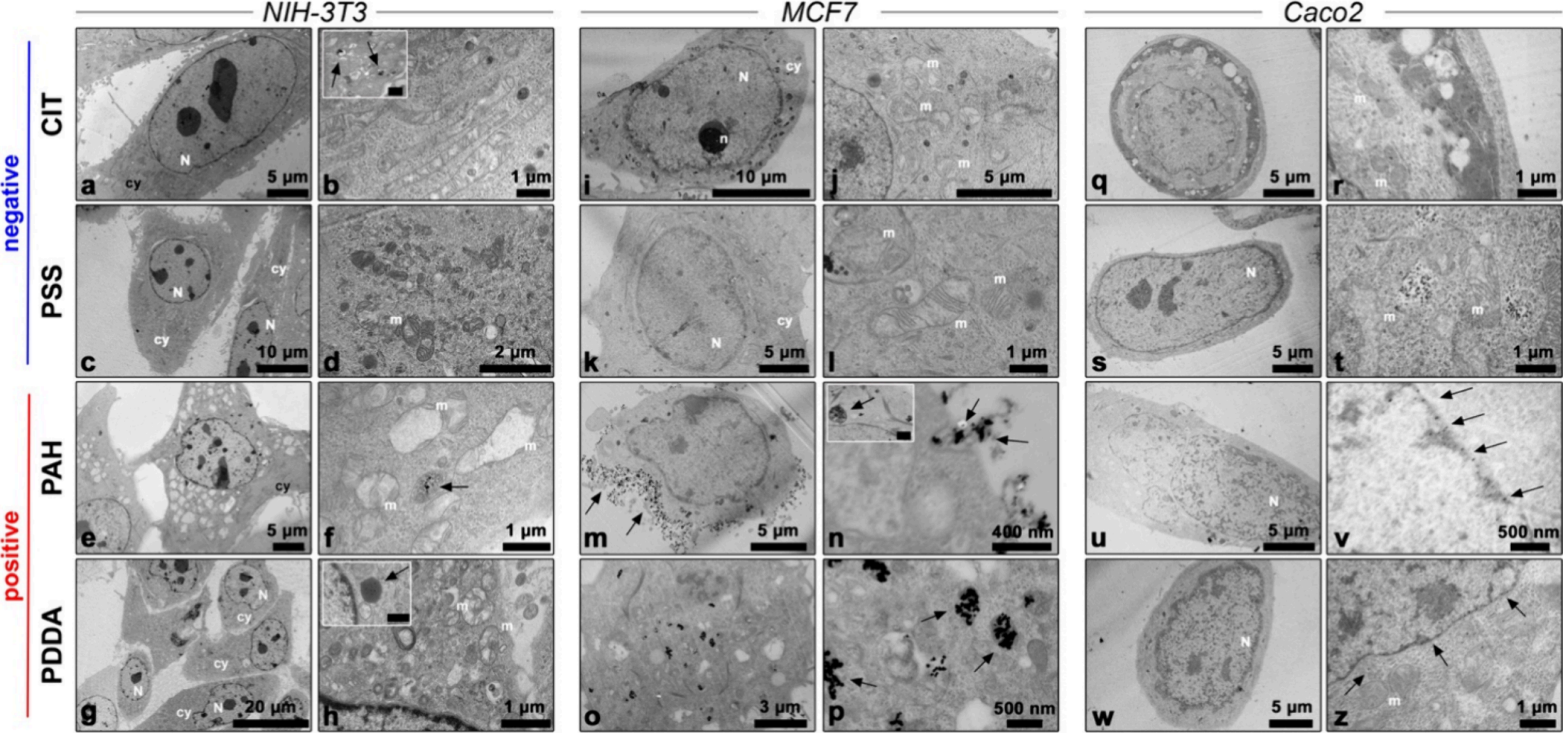
TEM images of ultrathin sections of NIH-3T3 (a–h), MCF7 (i–p), and Caco2 (q–z) cells treated for 6 h with 20 μg/mL citrate- (first row), PSS- (second row), PAH- (third row), and PDDA-coated (fourth row) AgNPs. Legend: nucleus (N), nucleoli (n), cytoplasm (cy), and mitochondria (m). Black arrows indicate the presence of nanoparticles. Scale bar in insets: 500 nm (b and h) and 1 μm (n).

Untreated MCF7 cells exhibited their typical round morphology, and the nuclei displayed nucleoli with homogeneous chromatin, free of any condensed areas (Figure S3c,d). The treatment of MCF7 cells with negatively charged AgNPs did not result in any significant changes at either the cellular or subcellular level after 6 h (Figure 4i–l), and notably, nanoparticles were not observed within the cells. However, even though we did not detect any significant cytotoxic activity of AgNPs-cit on MCF7 cells, even after 48 h of incubation (see previous section), some morphological changes in the shape and internal cristae of mitochondria were observed after 6 h of treatment with nanoparticles (Figure 4j). After 24 h of treatment, we were unable to detect negatively charged nanoparticles within the cells, but we did observe small areas of chromatin condensation (Figure S4i,k). Furthermore, cells treated with AgNPs-cit exhibited nuclei with irregular shapes and small, round mitochondria with partially damaged internal cristae (Figure S4i,j), while the cytoplasm of MCF7 treated with AgNPs-PSS was characterized by the presence of several small vesicles (Figure S4l).

MCF7 cells exposed to positively charged AgNPs for 6 h did not exhibit signs of morphological damage (Figure 4m,o). However, in contrast to negatively charged AgNPs, a high concentration of nanoparticles was found inside the cells, particularly within lysosomes (black arrows in Figure 4n,p). Notably, in cells treated with PAH-coated AgNPs, nanoparticles were also observed on the cell membrane (black arrows in Figure 4m), and there was evidence of their cellular uptake and internalization into endocytic vesicles (black arrows in Figure 4n). Even after 24 h of exposure, the morphology of MCF7 was still preserved, and clusters of AgNPs were clearly detected inside lysosomes of different sizes (black arrows in Figure S4n,p).

The untreated Caco2 cells showed a polygonal morphology characterized by a round and sizable nucleus that occupies a significant portion of the cytoplasmic volume and uncompromised cellular organelles (Figure S3e,f). After 6 h of treatment with negatively charged AgNPs, which were observed in the cytoplasm with apparently no specific localization, the cells showed preserved morphology (Figure 4q–t). However, the cytoplasm of those treated with citrate-coated AgNPs was characterized by numerous vacuoles (Figure 4r), while those treated with PSS-coated AgNPs showed a reduced density of mitochondria, also characterized by a few cristae (Figure 4t). After 24 h of treatment with negatively charged AgNPs, mitochondrial damage became even more evident. Most notably, small clusters of condensed chromatin were observed in the nucleus, indicating early events of apoptosis (Figure S4q–t). In contrast, after 6 h of treatment, positively charged AgNPs were detected not only in the surrounding cytoplasm but also in the nuclear envelope of Caco2 cells (black arrows in Figure 4v,z). The morphology of the cells was retained (Figure 4u,w), but the chromatin already showed a slight level of condensation (Figure 4v,z). After 24 h of treatment, clusters of nanoparticles were found in the cytoplasm and within empty vesicles (black arrows in Figure S4u–z) and, in the case of cells treated with PAH-coated AgNPs, necrosis was already observed (Figure S4v).

Overall, TEM observations reveal common behaviors of NPs across different cell settings, along with significant variations influenced by their surface charge. In accordance with what was observed in the uptake experiment (as discussed above), positively charged AgNPs exhibited greater cellular uptake compared with their negatively charged counterparts, with notable accumulation within lysosomes and the nuclear envelope. The difference was especially noticeable in MCF7 cells, where negatively charged NPs were not observed inside the cells. The treatment with both negative and positive AgNPs initially did not impair the cellular morphology, but cellular alterations became evident over time. Positively charged AgNPs induced necrosis, while negatively charged ones led to early signs of apoptosis, characterized by mitochondrial damage and chromatin condensation. The severity of mitochondrial damage differs between the two surface charges, with positively charged AgNPs causing more pronounced alterations. Notably, except for MCF7 cells, where negative AgNPs did not have such a noticeable effect, mitochondrial damage was a common feature induced by both positively and negatively charged AgNPs, suggesting a shared cytotoxic mechanism.

### 2.6 Protein Adsorption, Identification, and Classification

To study the role of surface charge in protein adsorption, AgNPs were incubated for 24 h with the cell growth media used in cell cultures, specifically fibroblast (NIH-3T3) and cancer (MCF7 and Caco2) cell media. The resulting NP-protein complexes were characterized by DLS, *ζ*-potential measurement, and UV–vis spectroscopy. Both the mean hydrodynamic diameter and the *ζ*-potential of the AgNPs underwent significant changes after incubation with the cell growth medium, irrespective of the medium type and surface charge, as shown in Figure 5a,b. The hydrodynamic diameter of AgNPs increased significantly when exposed to growth media containing serum proteins. This exposure also resulted in the “normalization” of the *ζ*-potential, which stabilized at values between −25 and −30 mV, regardless of the initial surface charge, as previously observed.^44,48^ These results demonstrate the adsorption of serum biomolecules from the growth media onto the surfaces of all AgNPs, irrespective of their coating and surface charge. It is noteworthy that the diameter of positively charged NPs undergoes a more pronounced increase compared with their negatively charged counterparts. This observation suggests that in addition to biomolecule adsorption, positively charged NPs tend to form aggregates when exposed to cell growth media. This tendency is further confirmed by the significant alteration of their absorption spectra profiles following incubation with cell growth media (Figure S5).^49,50^ On the other hand, when AgNPs were incubated with the cell growth media devoid of serum proteins (i.e., in serum-free conditions), the immediate aggregation of AgNPs, regardless of surface charge, occurs due to the high ionic strength, which drastically reduces the electrostatic repulsions between the nanoparticles.^50^ These results highlight how the formation of the protein corona is essential for stabilizing nanoparticles in the cellular medium, thus making them available to interact with cells.

**Figure 5:**
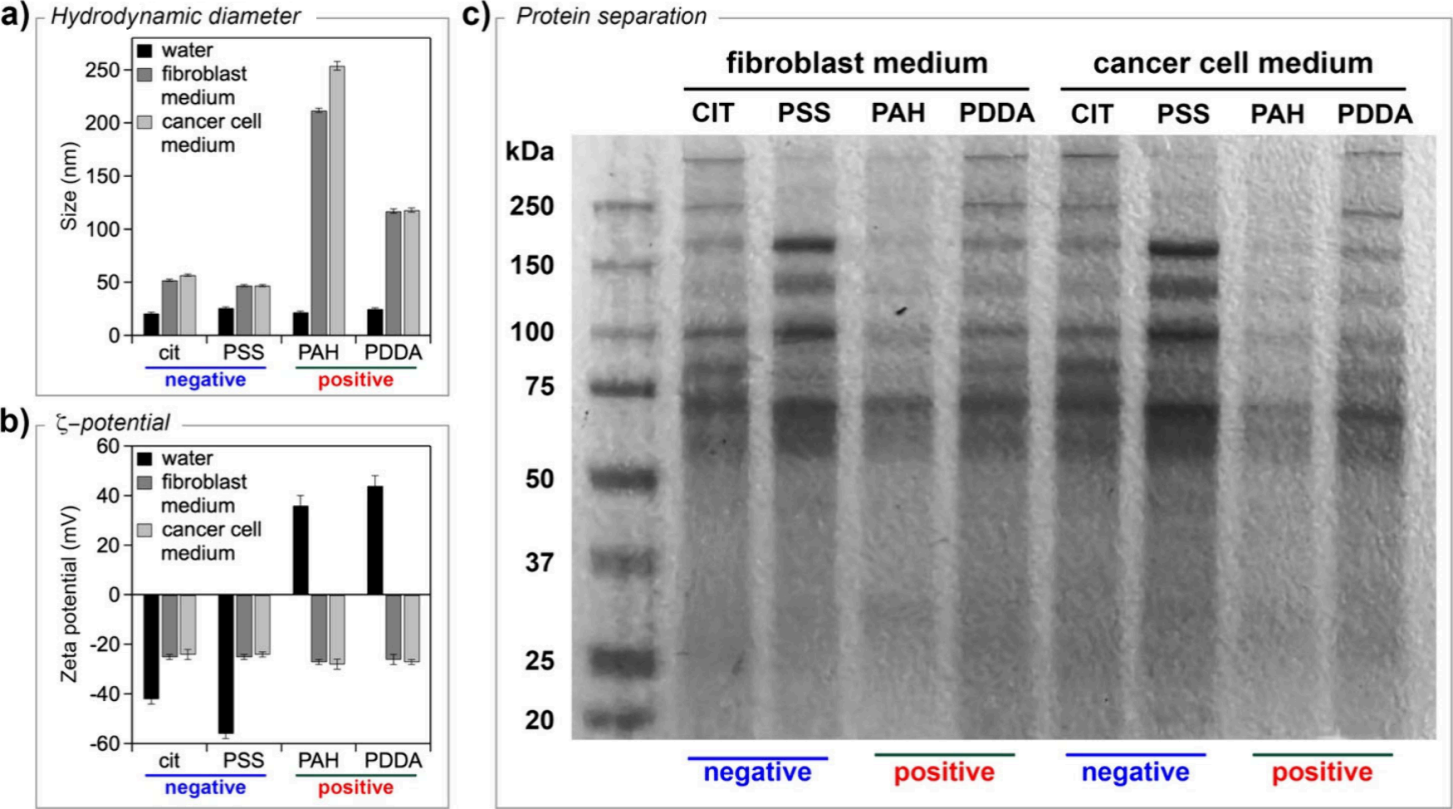
(a) Hydrodynamic diameter and (b) *ζ*-potential of AgNPs before (water) and after incubation in growth media: fibroblast (NIH-3T3) medium and cancer cell (MCF7 and Caco2) medium. Data are presented as mean ± SD and based on at least five independent measurements. (c) SDS-PAGE of biomolecules recovered from AgNPs after 24 h of incubation with fibroblast (NIH-3T3) medium and cancer cell (MCF7 and Caco2) medium. The molecular weight ladder is shown in lane 1.

To study the composition of the protein corona, the NP-protein complexes were separated by centrifugation and extensively washed, and the adsorbed biomolecules were isolated and separated using polyacrylamide gel electrophoresis (PAGE) coupled with protein staining (see the Experimental Section). As shown in Figure 5c, the protein corona of AgNPs is composed of a similar protein band pattern regardless of surface coating and growth medium, but the relative intensity of bands clearly changes, as revealed by densitometric analysis (Figure S6a,b). Notably, the total amount of biomolecules adsorbed on nanoparticle surfaces is quite similar regardless of the coating; however, in both cellular media, PAH-coated AgNPs showed the lowest level of adsorption (Figure S6c,d).

Overall, these results do not reveal significant differences in the protein corona across the various types of nanoparticles. Therefore, to gain a deeper understanding of the protein corona composition, we analyzed and identified the proteins using mass spectrometry and classified them through bioinformatics analysis. The complete list of proteins is summarized in Tables S1–S8. Overall, the number of identified proteins adsorbed on the NP surface varies from 100 to more than 200, depending on the surface coating and the cell growth medium employed.

In Figure 6a,b,d,e, the molecular weight (MW) and isoelectric point (pI) distributions of the identified adsorbed proteins are presented as a function of the nanoparticle surface coating and cell growth medium. Across all samples, more than 50% of identified proteins have a molecular weight below 60 kDa, but in the protein corona of positively charged NPs incubated with the cancer cell medium, there is a higher presence of high molecular weight proteins. In agreement with *ζ*-potential measurements (Figure 5b), the classification of proteins based on their pI shows that most of the corona proteins carry a negative charge at physiological pH (pI < 7), and in all cases, the largest fraction consists of proteins with a pI between 5 and 6. We also classified the proteins identified within the corona into six major groups based on their physiological functions, including the acute phase, coagulation, complement system, lipoproteins, tissue leakage, and other plasma components, in accordance with previous reports.^44,48,51–53^ As shown in Figure 6c,f, no drastic differences were observed in the distribution of protein functions in the protein corona of the different NPs. Therefore, regardless of the cell medium, the analysis of the MW, pI, and physiological function distributions of the adsorbed proteins suggests that there is no striking difference in the protein corona composition as the surface charge varies. However, the intersection analysis reveals that the protein coronas of the NPs exposed to the same cellular medium share a relatively low percentage of proteins. Specifically, AgNPs exposed to the NIH-3T3 medium shared 46 proteins, and those exposed to the cancer cell medium shared 54 proteins, representing 22% and 14% of all identified proteins, respectively (Figures S7 and S8). The shared proteins are summarized in Tables S9 and S10.

**Figure 6:**
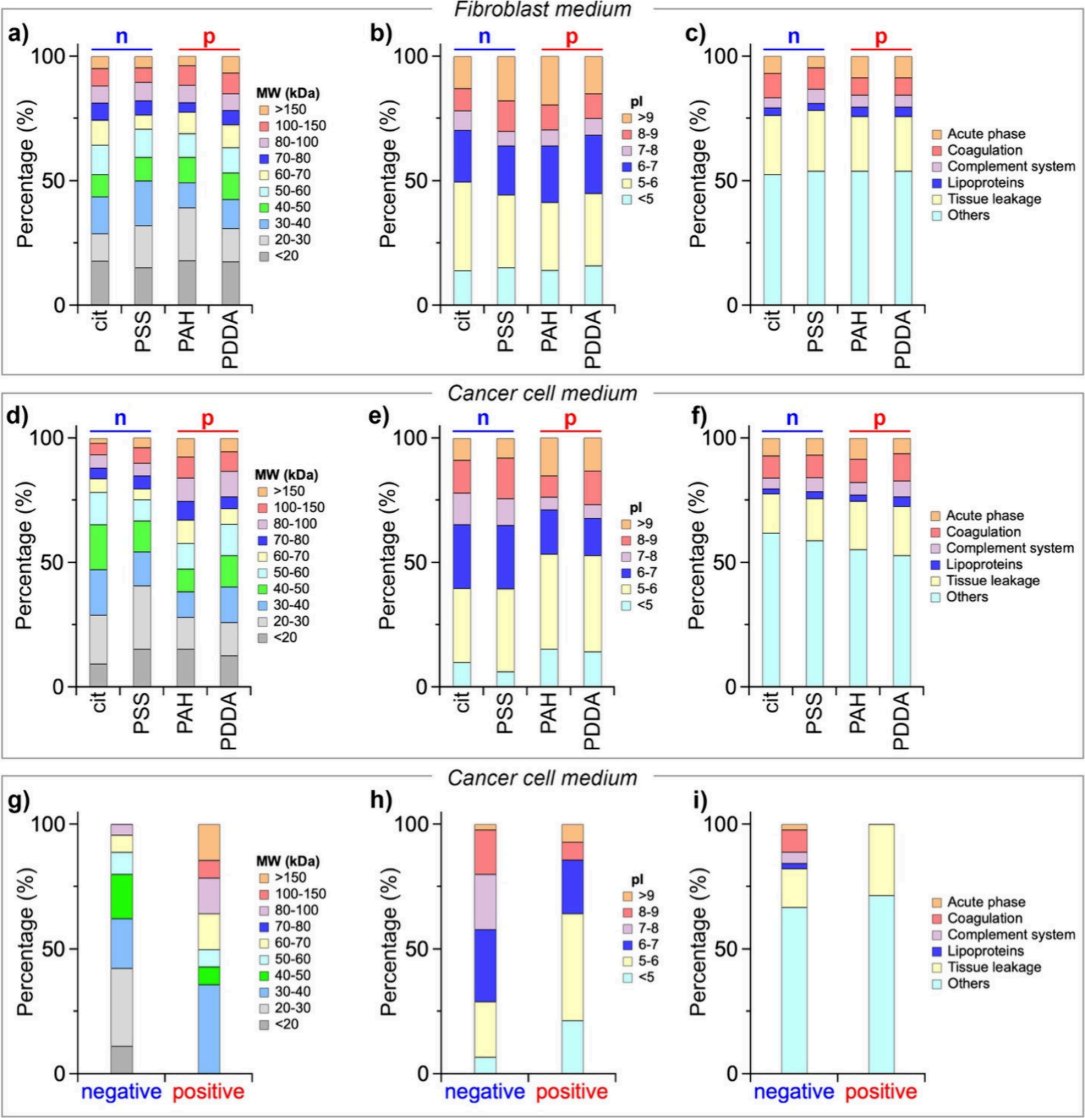
Bioinformatics classification of corona proteins: molecular weight (MW), isoelectric point (pI), and physiological function distribution of proteins recovered from AgNPs after 24 h of incubation with (a–c) fibroblast medium and (d–f) cancer cell medium. Surface charge of NPs: “n” denotes negatively charged NPs (AgNPs-cit and AgNPs-PSS) and “p” positively charged NPs (AgNPs-PAH and AgNPs-PDDA). (g–i) Distribution of proteins shared by the protein corona formed after 24 h of incubation with cancer cell medium on negatively charged NPs (AgNPs-cit and AgNPs-PSS) and positively charged NPs (AgNPs-PAH and AgNPs-PDDA).

As discussed previously, the surface charge of NPs plays a crucial role in their impact on MCF7 cells, with a notable disparity in cytotoxicity observed between positively and negatively charged NPs. However, the intersection analysis (Figure S8) also reveals that the proteins identified only in the protein corona of negatively charged NPs include 45 proteins (12% of the total proteins), whereas the proteins identified only in the protein corona of positively charged NPs include 14 proteins (4% of the total proteins). The shared proteins are summarized in Tables S11 and S12. By analyzing the distributions of MW, pI, and physiological functions of these shared proteins, some differences emerge. Compared to NPs with a negative surface charge, positively charged NPs preferentially adsorb higher MW proteins (Figure 6g). There is also a striking tendency for positively charged NPs to attract more acidic proteins, with a significant portion having a pI below 6 (Figure 6h). This result can be attributed to the tendency of positively charged NPs to become enriched with negatively charged proteins due to electrostatic interactions. On the other hand, unlike what was observed for whole coronas, differences in the distribution of physiological functions were also observed between the shared proteins of negatively charged NPs and positively charged ones (Figure 6i).

These results were reproducible and collectively confirm that protein corona formation is essential for stabilizing nanoparticles in physiological environments, limiting their aggregation and thus enabling their effective interaction with cells. However, the results demonstrate that protein adsorption on the surface of charged AgNPs appears to be largely a nonspecific process. Therefore, the biological activity of AgNPs is undoubtedly influenced by their surface charge, which is determined by the specific surface-coating molecules, particularly in relation to MCF7 cells. This suggests that the nanoparticle coating is sufficiently long-lived to play a critical role in mediating interactions between the nanoparticles and the cells. The biological response may also be indirectly affected by the composition of the protein corona; however, this relationship is not straightforward and appears to involve more complex mechanisms. This complexity may arise from the fact that we are primarily able to study what is known as the “hard corona”, while the interface that ultimately interacts with cells is the so-called “soft corona”.^54,55^ Further studies are needed to explore this aspect in greater detail.

## 3 Conclusions

In this study, we elucidated the interplay between surface charge and protein corona formation in governing the biological interactions of AgNPs. Our findings reveal that while protein corona formation is critical for stabilizing nanoparticles in physiological environments and ensuring their availability for cellular uptake, the surface charge predominantly determines their intracellular fate, biodistribution, and cytotoxic effects. Through comprehensive analysis of the effects of negatively and positively charged AgNPs across three cell lines, we demonstrated that the surface charge significantly influences the cytotoxic activity of AgNPs, and observations from fluorescence and electron microscopy complemented by optical emission spectroscopy further reveal that surface charge plays a crucial role in the biodistribution and cellular uptake of these nanoparticles. Moreover, our results show that positively charged AgNPs exhibit greater cytotoxicity and higher cellular uptake compared with their negatively charged counterparts, particularly in MCF7 cells. This behavior is likely driven by electrostatic interactions between the positively charged AgNPs and negatively charged cell membranes, prompting nanoparticle uptake. Enhanced internalization results, as revealed by ultrastructural studies, in greater accumulation of positively charged AgNPs in intracellular organelles such as lysosomes and mitochondria, leading to pronounced cellular damage. In contrast, negatively charged AgNPs primarily induce early apoptosis, with less severe mitochondrial damage. On the other hand, the analysis of the protein corona using gel electrophoresis, mass spectrometry, and gene ontology analysis reveals no evidence of preferential protein adsorption based on the surface charge of the AgNPs. This indicates that while surface charge clearly promotes the formation of the protein corona—stabilizing the nanoparticles and preventing their aggregation in the cellular medium, thereby ensuring their availability for interactions with cells—the absorption process does not appear to be specific to the type of surface charge. Therefore, it is the surface charge that ultimately plays a direct role in interactions with cells rather than the result of specific protein enrichment based on surface charge.

Overall, our results unveil that despite the shielding effect of the protein corona, the surface charge of the nanoparticles remains a key determinant of their cellular interactions and intracellular behavior, raising important questions about the mechanisms by which surface charge exerts its influence in the presence of the protein corona. However, our analysis focused on the so-called “hard corona”, the stable protein layer tightly bound to the nanoparticle surface, and further efforts will be needed to investigate the role of the “soft corona”, the more dynamic and loosely associated layer of proteins, in the overall interactions at the nano–bio interface. These insights emphasize the importance of delving deeper into the mechanistic interplay between surface charge and protein corona formation to advance the design of engineered nanoparticles with optimized safety profiles and enhanced therapeutic efficacy.

## 4 Experimental Section

### 4.1 Materials

Silver nitrate (AgNO_3_), sodium citrate (C_6_H_5_O_7_Na_3_), tannic acid (C_76_H_52_O_46_), poly­(allylamine hydrochloride) (PAH; MW = 17 500 g/mol), poly­(diallyl­dimethyl­ammonium chloride) (PDDA; MW = 100 000–200 000 g/mol), poly­(sodium 4-styrene­sulfonate) (PSS; MW = 70 000 g/mol), MEM Non-Essential Amino Acids (NEAA), Dulbecco’s phosphate buffered saline (DPBS), and 3-(4,5-dimethyl-2-thiazolyl)-2,5-diphenyl­tetra­zolium bromide (MTT) were purchased from Merck and used without further purification. All aqueous solutions were prepared with deionized water obtained by using an ultrafiltration system (Milli-Q, Millipore) with a measured resistivity above 18 MΩ. Dulbecco’s modified Eagle medium (DMEM) and fetal bovine serum (FBS) were purchased from Gibco.

### 4.2 Synthesis of Citrate-Coated AgNPs

Citrate-stabilized AgNPs were prepared following a slightly modified method reported elsewhere.^41^ In brief, 100 mL of an aqueous solution of sodium citrate (5 mM) and tannic acid (0.025 mM) was refluxed, and an aqueous solution of silver nitrate (1 mL, 25 mM) was added quickly. Then, the reaction mixture was refluxed for 15 min, resulting in a bright yellow colloidal silver solution, and was then left to cool to room temperature. The aqueous suspension of AgNPs was purified by two rounds of centrifugation (30 000*g* for 1 h) and resuspension in 10 mL of an aqueous solution of sodium citrate (2 mM).

### 4.3 Characterization of AgNPs

UV–vis spectra were recorded on a Jasco V-550 UV/Vis spectrophotometer. Dynamic light scattering (DLS) and *ζ*-potential measurements were performed in phosphate buffer (1 mM, pH 7) and KCl (1 mM) or deionized water on a NanoBrook Omni Particle Size Analyzer (Brookhaven Instruments Corporation, USA) equipped with a 35 mW red diode laser (nominal 640 nm wavelength). AgNPs were characterized using transmission electron microscopy (TEM) with an FEI Tecnai F20 ST instrument equipped with a dispersion microanalysis of energy (EDS) and the scanning transmission electron microscopy (STEM) accessory. TEM samples were prepared by drop casting nanoparticle solutions onto a holey carbon-coated gold grid and dried at 80 °C. AgNPs coated with polyelectrolytes were stained with phosphotungstic acid (2 wt %, pH adjusted to 7 by adding NaOH) according to a previously reported procedure.^56^ The average size and size distribution of citrate-stabilized AgNPs were measured by counting more than 800 particles. The TEM images were taken in phase contrast mode and selected area electron diffraction (SAED). STEM pictures were recorded using high angle annular dark field (HAADF) detectors: in this imaging mode, the intensity *I* is proportional to *Z* ^1.7^ *t*, where *Z* is the mean atomic number and *t* is the thickness of the specimen. Silver concentrations were measured by inductively coupled plasma optical emission spectroscopy using an ICP-OES 5100 vertical dual view apparatus (Agilent Technologies, Santa Clara, CA, USA).

### 4.4 Positively Charged AgNPs (AgNPs-PAH and AgNPs-PDDA)

The as-prepared AgNPs (1 mL) were transferred in deionized water (2 mL) and added dropwise to PAH or PDDA (1 g/L, 2 mL) in NaCl (1 mM) aqueous solution under gentle stirring. The excess of polymer was removed by two rounds of centrifugation (2000*g* for 60 min) and resuspension in deionized water, recovering approximately 85% of the nanoparticles.

### 4.5 Negatively Charged AgNPs (AgNPs-PSS)

The as-prepared AgNPs-PAH (1 mL) were diluted with deionized water (1 mL) and added dropwise to PSS (5 g/L, 2 mL) in NaCl (1 mM) aqueous solution under gentle stirring. The excess of polymer was removed by two rounds of centrifugation (2000*g* for 60 min) and resuspension in deionized water, recovering approximately 90% of the nanoparticles.

### 4.6 Serum Protein Adsorption

AgNPs were mixed in a 1:14 volume ratio with complete medium (with or without 0.1 mM MEM NEAA) and incubated overnight at 37 °C (final silver concentration = 20 μg/mL). The nanoparticles were purified by three rounds of centrifugation (13 000*g* for 30 min at 4 °C) and resuspension in phosphate buffer (1 mM, pH 7) and KCl (1 mM).

### 4.7 Polyacrylamide Gel Electrophoresis (PAGE)

AgNPs were transferred by centrifugation (13 000*g* for 20 min at 4 °C) in Tris-Cl (10 mM, pH 7.4) and then resuspended in protein loading buffer (62.5 mM Tris-HCl pH 6.8, 10% (v/v) glycerol, 1% LDS, 0.0045% bromophenol blue, 50 mM DTT) and boiled at 100 °C for 5 min. Nanoparticles were removed by centrifugation (13 000*g* for 30 min at 4 °C), and the supernatants, along with a molecular weight ladder (Bio Rad), were loaded on 10% SDS-PAGE and resolved at 100 V for 60 min. The gel was fixed with a solution of 25% isopropyl alcohol and 10% glacial acetic acid for 60 min and stained with colloidal Coomassie Blue G-250 for 2 h. Gel densitometry was performed using ImageJ 1.54g.

### 4.8 Mass Spectrometry Analysis and Protein Identification

As reported above, protein separation was performed by PAGE using precast polyacrylamide gel (Bio Rad). The resulting gel was stained overnight with colloidal Coomassie Blue G-250, and bands were sliced and processed as previously described.^38^ After tryptic digestion, peptides were completely dried in a SpeedVac system and then resuspended in 20 μL of a 97:3:2 mixture of water:acetonitrile:​formic acid for mass spectrometry analysis. The analysis was performed on an Ultimate 3000 nano UHPLC coupled to an Exploris 480 Hybrid Quadrupole-Orbitrap Mass Spectrometer by means of an Easy-Nano electrospray ion source (Thermo Fisher Scientific). Each sample (1μL) was injected onto a 0.3 × 5 mm Pepmap100 C18 5 μm cartridge kept at 45 °C with a 30 μL/min flow rate using 0.1% formic acid in water solution. The loading/washing step was performed for 2 min, and then the preconcentrated peptides were directed toward an Easy-Spray PepMap C18 150 mm 75 μm ID 3 μm ps column peptide separation, with a 250 nL/min flow rate. The mobile phase components were (A) 0.1% formic acid in water and (B) 0.1% formic acid in acetonitrile:water (80:20). The chromatographic separation was performed at 35 °C with a linear gradient from 6% to 31% B in 30 min and from 31% to 50% B in 5 min. The gradient was then raised to 95% B for a 5 min washing step and then rapidly lowered to 6% B for the final reconditioning step pending next injection; the total run time was 70 min. The electrospray source was used in positive ionization mode without a sweep gas flow (0), with an ion transfer tube temperature at 275 °C and a spray voltage of 1600 V. Mass spectrometric detection of peptides was performed using full scan data-dependent MS^2^ experiments. Full MS spectra were acquired at 120 000 FWHM resolution (for *m*/*z* 200), detecting ions within the 375 to 1700 scan range with the normalized AGC target set at 300%; filters for monoisotopic peak determination (peptides), intensity (>10^4^), charge state (2 to 5), and dynamic exclusion (1 time selection, 45 s exclusion duration) were used to select the top 20 dependent MS^2^ scans. MS^2^ spectra were acquired at 15 000 FWHM resolution (for *m*/*z* 200) with the normalized AGC target set to 50% and the isolation width and offset being respectively 1.5 and 0.4 and with the HCD collision energy at 30%. Raw MS/MS data were converted by msConvert ProteoWizard (v.3.0.19239) in .mgf files using default settings and uploaded to Mascot Daemon (Mascot server v.2.7.0) for MS/MS Ion Search. The search was performed using the SwissProt database (2018_05; 557 491 sequences) restricted to Bos taurus, and the reverse decoy database (cRAP) was added to calculate the false discovery rate (FDR) due to random match. Furthermore, parameters for identification included the following: (i) trypsin as an enzyme with 1 of maximum missed cleavage; (ii) mass error tolerances for precursor and fragment ions set to 10 ppm and 0.02 Da, respectively; (iii) peptide charge (2+, 3+, 4+); and (iv) carbamidomethyl cysteine (C), set as fixed modification, while deamidation of asparagine and glutamine (NQ) and oxidation of methionine (M) were considered as dynamic modification. Data from the same original sample were merged. The FDR for protein identification based on the sequence homology was set to 1%.

### 4.9 Cell Cultures

Human breast cancer cells (ATCC), MCF7, and human colon cancer cells (Merck), Caco2, cells were cultured under standard conditions in DMEM supplemented with 10% (v/v) heat-inactivated FBS, 2 mM l-glutamine, 100 U mL^–1^ penicillin, and 100 U mL^–1^ streptomycin in a humidified incubator set at 37 °C with 5% CO_2_. Mouse embryonic fibroblast cells (Merck), NIH-3T3, were cultured under standard conditions in DMEM supplemented with 10% (v/v) heat-inactivated FBS, 2 mM l-glutamine, 0.1 mM MEM Non-Essential Amino Acids (NEAA), 100 U mL^–1^ penicillin, and 100 U mL^–1^ streptomycin in a humidified incubator set at 37 °C with 5% CO_2_. Cells were seeded in 96-, 24-, 12-, and 6-well plates and grown for 24 h before exposure to nanoparticles. For experimental controls, the cell culture medium was diluted with deionized water (vehicle) to ensure that dilution of the medium by a solution of nanoparticles has no impact on the cell performance.

### 4.10 Actin and Nucleus Staining

Cells were seeded in 24-well plates and treated with AgNPs for 24 or 48 h. The cells were fixed with 4% paraformaldehyde in DPBS and washed with DPBS. They were then permeabilized with 0.001% Triton-X 100 (Merck Millipore). The cells were labelled with TRITC-conjugated phalloidin (FAK100, Merck Millipore) for 1 h, followed by rinses with DPBS. Nuclear counterstaining was performed by incubation with DAPI (4′,6-diamidino-2-phenylindole, Merck Millipore) for 3 min, followed by rinses with DPBS. The samples were examined using a Nikon Eclipse 80i microscope equipped for fluorescence analysis.

### 4.11 Cell Viability Assay

Cell viability was determined by MTT (3-(4,5-dimethyl-2-thiazolyl)-2,5-diphenyl­tetra­zolium bromide) assay measuring the intracellular reduction of tetrazolium salts into purple formazan by viable cells.^57^ Briefly, the cells were seeded in 96-well plates and treated with AgNPs for 24 or 48 h under standard conditions. After incubation, the medium with or without AgNPs was discarded, and the cells were washed two times with 100 μL of DPBS. Afterward, 100 μL of sterile MTT solution (1 mg mL^–1^ in DPBS) was added to each well and incubated for 1 h at 37 °C with 5% CO_2_. Subsequently, the medium was discarded, and 200 μL of DMSO was added to each sample to solubilize formazan crystals. Optical density (OD) was read on a microplate reader (Thermo Scientific Varioskan Flash Multimode Reader) at 550 nm as a working wavelength and 640 nm as a reference. Cell viability was calculated as the proportion of the mean OD of the replicated wells relative to that of the control.

### 4.12 In Vitro Uptake of AgNPs

The uptake of AgNPs by cells was quantified using inductively coupled plasma optical emission spectroscopy (ICP-OES). Cells were seeded in 12-well plates and treated with AgNPs for 16 h. After exposure, the medium was discarded, and the cells were washed three times with a saline solution to remove residual NPs. Then, the cells were treated with trypsin-EDTA 1×, neutralized with DPBS, counted, washed with deionized water, and collected by centrifugation. The recovered pellets were digested overnight with a mixture of hydrogen peroxide and nitric acid (HNO_3_:H_2_O_2_ = 1:1) and then diluted with deionized water. The concentration of silver was measured by ICP-OES titration at λ_Ag_ = 328.068 nm. Operating conditions of the ICP-OES are listed here: RF power, 1200 W; plasma Ar flow rate, 12 L/min; nebulizer Ar flow rate, 0.70 L/min; uptake time, 28 s; stabilization time, 15 s. A series of silver standard solutions (5, 2.5, 1.0, 0.5, 0.25, 0.05, and 0 ppm) in HNO_3_ 65%/H_2_O_2_ 30%/Milli-Q water (1:1:8 mixing ratio by volume) were prepared to obtain a calibration curve used to determine the silver amount taken up by the cells in each sample. The concentration reported for each sample is the mean value of five different measures.

### 4.13 Annexin V Staining

Cells were seeded in 12-well plates and treated with AgNPs for 24 or 48 h. Apoptosis analyses were then performed through an Annexin V-FITC Apoptosis Detection kit (eBioscience, Thermo Fisher Scientific, Waltham, MA, USA), according to the manufacturer’s instructions. Briefly, after the indicated treatments, cells were collected and washed twice in PBS and stained in binding buffer with Annexin V-FITC. Then, cells were washed again and resuspended in binding buffer with propidium iodide. Analyses were performed on an Attune NxT flow cytometer (Thermo Fisher Scientific, USA) with the appropriate software (Attune Cytometric Software version 5.0). At least 10 000 events per sample were acquired.

### 4.14 Transmission Electron Microscopy (TEM) Analysis

Cells were seeded on cover glasses in 6-well plates and treated with AgNPs for 6 or 24 h. At the end of each treatment, cells were washed in PBS to remove any cell debris and unbinding nanoparticles; then, they were fixed with 2.5% (v/v) glutaraldehyde in 0.1 M cacodylate buffer for 2 h and 30 min at 4 °C. After three washes in 0.15 M cacodylate buffer, samples were postfixed with a solution of 1% osmium tetroxide in 0.1 M cacodylate buffer for 30 min at room temperature. Following three washes in 0.15 M cacodylate buffer, cells were dehydrated in an ascending acetone series and finally embedded in epoxy resins (Sigma-Aldrich, USA). Ultrathin slices of 100 nm were stained by uranyl acetate solution, carbon coated, and then observed with a NanoSEM 450 (FEI Company, Eindhoven, The Netherlands) field emission gun (FEG) scanning electron microscope (SEM) working in TEM modality (STEM), at an accelerating voltage of 30 kV.

### 4.15 Bioinformatics Analysis

Visual analysis was conducted on the intersection of proteins identified on the surface of AgNPs after incubation in growth media and purification, using the UpSetR package.^58^ The isoelectric point (pI, Bjellqvist model) and molecular weight (MW) of proteins were obtained from the proteome pI database.^59^

### 4.16 Statistical Analysis

All data represent the mean ± standard deviation (SD) of at least four independent experiments. Statistical significance was determined using a one-way analysis of variance with Tukey’s test for multiple comparisons using Origin 8 software (OriginLab Corporation). Differences were considered significant when *p* < 0.005 and *p* < 0.001.

## Supplementary Material



## Notes

Cell viability; fluorescence micrographs of cells treated with vehicle solution; TEM images of ultrathin sections of cells treated with vehicle solution and those treated for 24 h with AgNPs; UV–vis absorption spectra of AgNPs after incubation in media; profile of the SDS-PAGE lanes; UpSet plots of the intersection of absorbed proteins onto the surface of AgNPs; the proteins recovered from the protein corona of AgNPs; and the proteins shared by the different protein coronas (PDF)
