# Supplementary material for: Surface Charge Overrides Protein Corona Formation in Determining the Cytotoxicity, Cellular Uptake, and Biodistribution of Silver Nanoparticles
Source: ACS Appl Bio Mater. 2025 May 21;8(6):5032–43. doi: 10.1021/acsabm.5c00392 (PMC12175132; doi:10.1021/acsabm.5c00392)
Supplement: Supplementary file 1 [file mt5c00392_si_001.pdf]

Supporting Information

Surface charge overrides protein corona formation in determining the cytotoxicity, cellular uptake, and biodistribution of silver nanoparticles

*Marianna Barbalinardo,[a] Francesca Chiarini,[b] Gabriella Teti,[c] Francesca Paganelli,[c] Elisa Mercadelli,[d] Andrea Bartoletti,[d] Andrea Migliori,[e] Manuela Piazzi,[f] Jessika Bertacchini,[g] Paola Sena,[g] Alessandra Sanson,[d] Mirella Falconi,[c] Carla Palumbo,[b] Massimiliano Cavallini[a] and Denis Gentili[a]**

a) Consiglio Nazionale delle Ricerche, Istituto per lo Studio dei Materiali Nanostrutturati (CNR-ISMN), via P. Gobetti 101, 40129 Bologna, Italy

b) Department of Biomedical, Metabolic and Neural Sciences, Section of Human Morphology, University of Modena and Reggio Emilia, via del Pozzo 71, 41124 Modena, Italy

c) Department of Biomedical and Neuromotor Sciences, University of Bologna, via Irnerio 48, 40126, Bologna, Italy

d) Consiglio Nazionale delle Ricerche, Istituto di Scienza, Tecnologia e Sostenibilità per lo Sviluppo dei Materiali Ceramici (ISSMC), via Granarolo 64, 48018, Faenza, Italy

e) Consiglio Nazionale delle Ricerche, Istituto di Genetica Molecolare (CNR-IGM), via Di Barbiano 1/10, Bologna, Italy

f) Department of Surgery, Medicine Dentistry and Morphological Sciences with Interest in Transplant, University of Modena and Reggio Emilia, via del Pozzo 71, 41124 Modena, Italy

g) Department of Medical and Surgical Sciences, University of Bologna, via Irnerio 48, 40126, Bologna, Italy

Corresponding Author

* Denis Gentili - Consiglio Nazionale delle Ricerche, Istituto per lo Studio dei Materiali Nanostrutturati (CNR-ISMN), 40129 Bologna, Italy; orcid.org/0000-0002-7599-2804; Email: denis.gentili@cnr.it

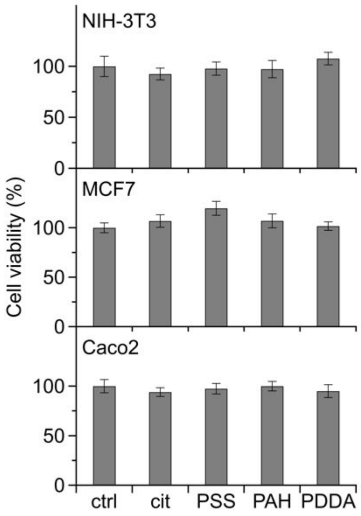

Figure S1. (a) Cell viability of NIH-3T3, MCF7 and Caco2 cells treated for 48 hours with supernatant liquid obtained after centrifugation of AgNPs. Data represent the mean ± SD and are plotted as percentage referred to control samples (ctrl).

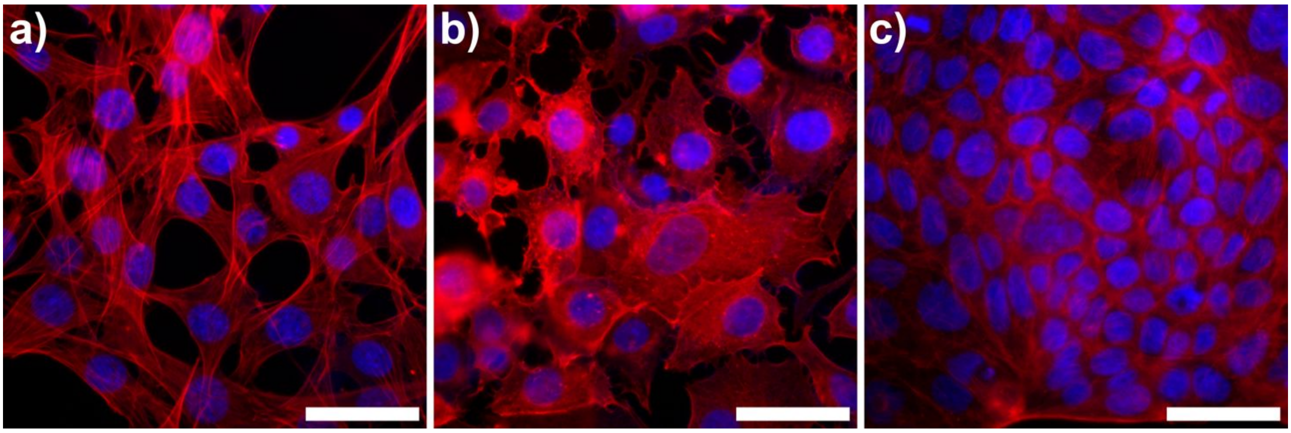

Figure S2. Fluorescence micrographs of (a) NIH-3T3, (b) MCF7 and (c) Caco2 cells labeled specifically for actin (red) and the nucleus (blue) and treated with vehicle solution for 48 hours.

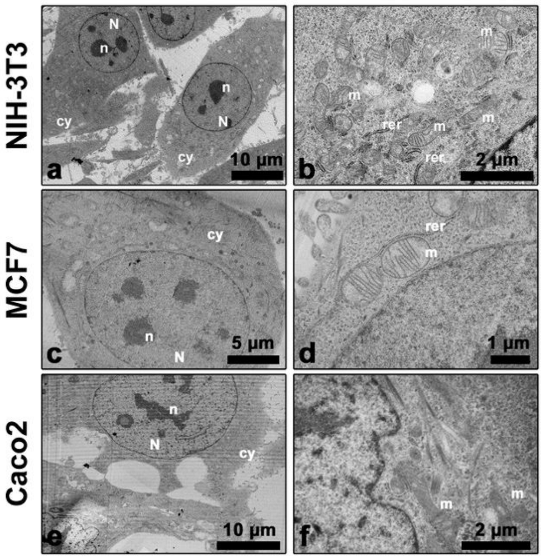

Figure S3. TEM images of ultrathin sections of NIH-3T3 (a-b), MCF7 (c-d) and Caco2 (e-f) cells treated for 24h with vehicle solution. Legend: Nucleus (N), nucleoli (n), cytoplasm (cy), mitochondria (m) and rough endoplasmic reticulum (rer).

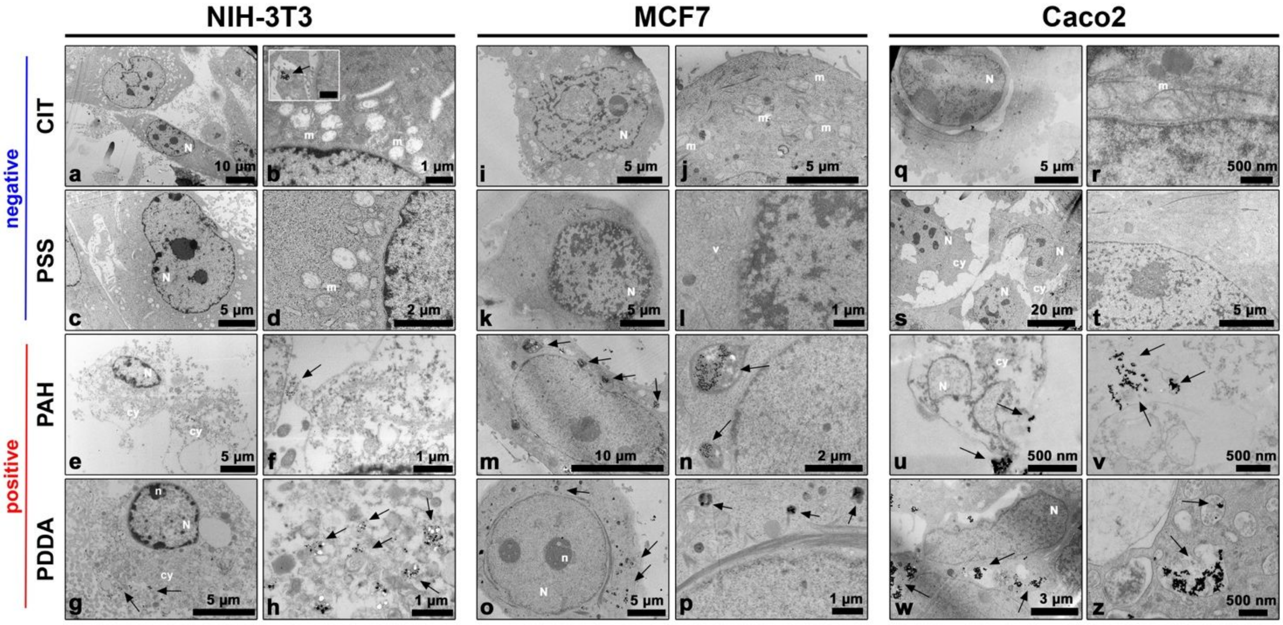

Figure S4. TEM images of ultrathin sections of NIH-3T3 (a-h), MCF7 (i-p) and Caco2 (q-z) cells treated for 24h with 20 µg/mL of citrate- (first row), PSS- (second row), PAH- (third row) and PDDA-coated (fourth row) AgNPs. Legend: Nucleus (N), nucleoli (n), cytoplasm (cy), vesicles (v) and mitochondria (m). Black arrows indicate the presence of nanoparticles. Inset: scale bar 1 µm.

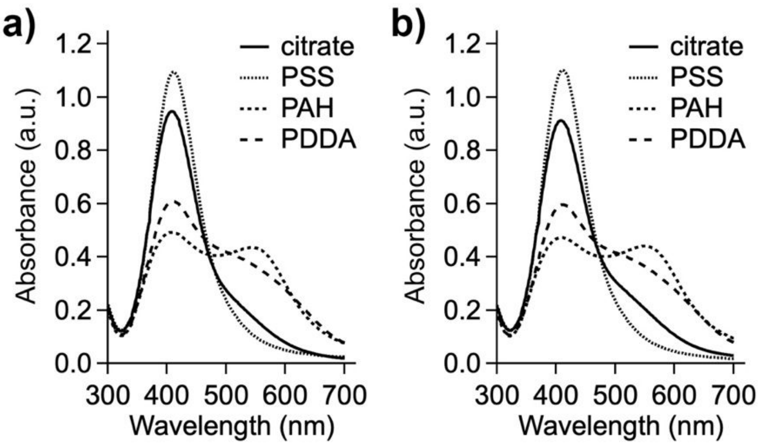

Figure S5. UV/Vis absorption spectra of AgNPs after 24 hours of incubation in complete medium of (a) normal cell line (NIH-3T3) and (b) human cancer cell lines (MCF7, Caco2).

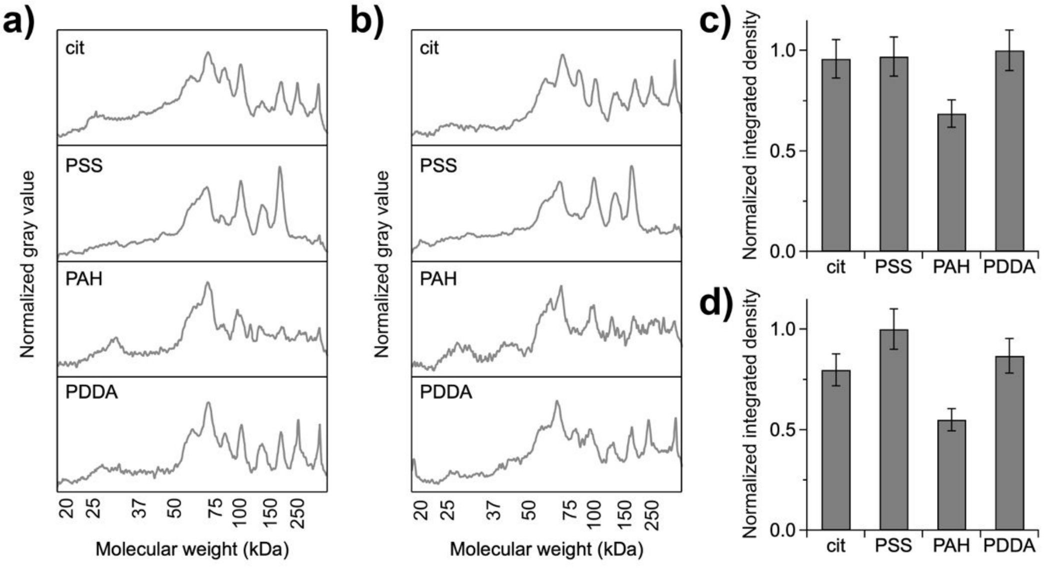

Figure S6. Profile of the SDS-PAGE lanes (Figure 5c) of biomolecules recovered from AgNPs after 24 h incubation with (a) NIH-3T3 or (b) human cancer cell medium. Integrated density of the SDS-PAGE lanes (Figure 5c) of biomolecules recovered from AgNPs after 24 h incubation with (c) NIH-3T3 or (d) human cancer cell medium.

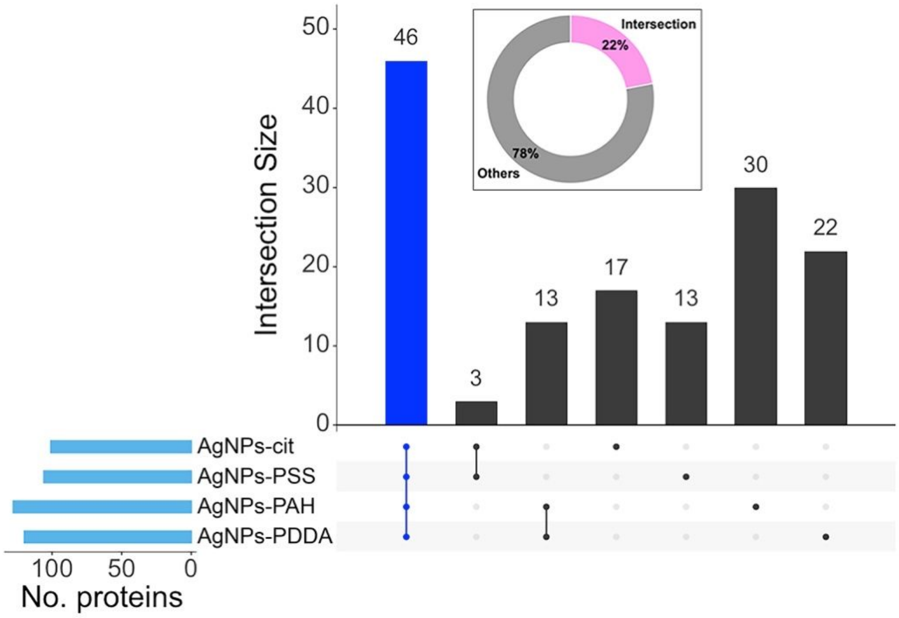

Figure S7. UpSet plot of the intersection of absorbed proteins onto the surface of the different AgNPs after 24 hours of incubation in complete medium of NIH-3T3 cells. The left bar figure shows the total number of absorbed proteins for the different AgNPs. The inset shows the percentage of proteins shared by all AgNPs.

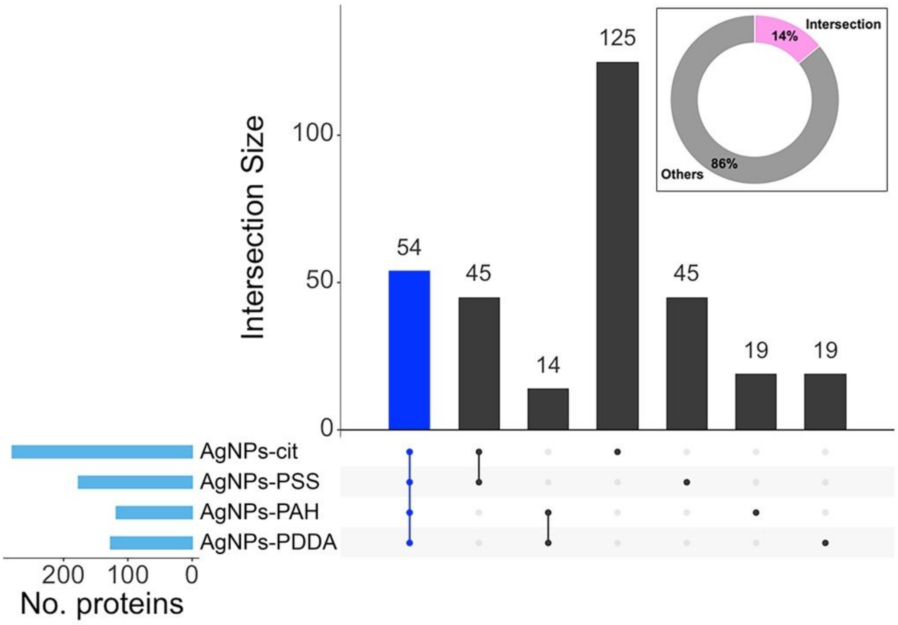

Figure S8. UpSet plot of the intersection of absorbed proteins onto the surface of the different AgNPs after 24 hours of incubation in complete medium of human cancer cells. The left bar figure shows the total number of absorbed proteins for the different AgNPs. The inset shows the percentage of proteins shared by all AgNPs.

Table S1: List of proteins recovered from citrate-coated AgNPs after 24 h incubation with NIH-3T3 medium

Abbreviation	Name
1433B_BOVIN	14-3-3 protein beta/alpha
A1AT_BOVIN	Alpha-1-antiproteinase
A2AP_BOVIN	Alpha-2-antiplasmin
A2MG_BOVIN	Alpha-2-macroglobulin
ACTA_BOVIN	Actin, aortic smooth muscle
ACTB_BOVIN	Actin, cytoplasmic 1
ALBU_BOVIN	Serum albumin
ANT3_BOVIN	Antithrombin-III
ANXA2_BOVIN	Annexin A2
APOA1_BOVIN	Apolipoprotein A-I
APOE_BOVIN	Apolipoprotein E
ARF1_BOVIN	ADP-ribosylation factor 1
ATPA_BOVIN	ATP synthase subunit alpha, mitochondrial
ATPB_BOVIN	ATP synthase subunit beta, mitochondrial
BIP_BOVIN	Endoplasmic reticulum chaperone BiP
CALD1_BOVIN	Non-muscle caldesmon (Fragment)
CASA1_BOVIN	Alpha-S1-casein
CFAB_BOVIN	Complement factor B
CFAH_BOVIN	Complement factor H
CH10_BOVIN	10 kDa heat shock protein, mitochondrial
CH60_BOVIN	60 kDa heat shock protein, mitochondrial
CLUS_BOVIN	Clusterin
CNN3_BOVIN	Calponin-3
CO1A1_BOVIN	Collagen alpha-1(I) chain
CO3_BOVIN	Complement C3
CO4_BOVIN	Complement C4 (Fragments)
EF1A1_BOVIN	Elongation factor 1-alpha 1
EF1G_BOVIN	Elongation factor 1-gamma
ENOA_BOVIN	Alpha-enolase
ENPL_BOVIN	Endoplasmin
EZRI_BOVIN	Ezrin
F13A_BOVIN	Coagulation factor XIII A chain (Fragment)
FA5_BOVIN	Coagulation factor V
FETUA_BOVIN	Alpha-2-HS-glycoprotein
FIBA_BOVIN	Fibrinogen alpha chain
FINC_BOVIN	Fibronectin
G3P_BOVIN	Glyceraldehyde-3-phosphate dehydrogenase

GELS_BOVIN	Gelsolin
H2A1_BOVIN	Histone H2A type 1
H2A2C_BOVIN	Histone H2A type 2-C
H2B1K_BOVIN	Histone H2B type 1-K
H4_BOVIN	Histone H4
HBA_BOVIN	Hemoglobin subunit alpha
HBBF_BOVIN	Hemoglobin fetal subunit beta
HGFL_BOVIN	Hepatocyte growth factor-like protein
HMGB2_BOVIN	High mobility group protein B2
HNRPK_BOVIN	Heterogeneous nuclear ribonucleoprotein K
HS71A_BOVIN	Heat shock 70 kDa protein 1A
HS71L_BOVIN	Heat shock 70 kDa protein 1-like
HS90A_BOVIN	Heat shock protein HSP 90-alpha
HSP72_BOVIN	Heat shock-related 70 kDa protein 2
HSP7C_BOVIN	Heat shock cognate 71 kDa protein
IBP2_BOVIN	Insulin-like growth factor-binding protein 2
IPSP_BOVIN	Plasma serine protease inhibitor
ITIH1_BOVIN	Inter-alpha-trypsin inhibitor heavy chain H1
ITIH3_BOVIN	Inter-alpha-trypsin inhibitor heavy chain H3
ITIH4_BOVIN	Inter-alpha-trypsin inhibitor heavy chain H4
K1C10_BOVIN	Keratin, type I cytoskeletal 10
K2C5_BOVIN	Keratin, type II cytoskeletal 5
KNG1_BOVIN	Kininogen-1
LASP1_BOVIN	LIM and SH3 domain protein 1
LDHB_BOVIN	L-lactate dehydrogenase B chain
LEG1_BOVIN	Galectin-1
LUM_BOVIN	Lumican
MAP4_BOVIN	Microtubule-associated protein 4
MDHC_BOVIN	Malate dehydrogenase, cytoplasmic
MYH10_BOVIN	Myosin-10
MYL6_BOVIN	Myosin light polypeptide 6
MYL9_BOVIN	Myosin regulatory light polypeptide 9
NDKB_BOVIN	Nucleoside diphosphate kinase B
PDIA3_BOVIN	Protein disulfide-isomerase A3
PLMN_BOVIN	Plasminogen
PPIA_BOVIN	Peptidyl-prolyl cis-trans isomerase A
PPIB_BOVIN	Peptidyl-prolyl cis-trans isomerase B
PRDX1_BOVIN	Peroxiredoxin-1
RL14_BOVIN	60S ribosomal protein L14
RL7_BOVIN	60S ribosomal protein L7

RLA2_BOVIN	60S acidic ribosomal protein P2
ROA2_BOVIN	Heterogeneous nuclear ribonucleoproteins A2/B1
RS13_BOVIN	40S ribosomal protein S13
RS28_BOVIN	40S ribosomal protein S28
SAHH_BOVIN	Adenosylhomocysteinase
SPA31_BOVIN	Serpin A3-1
SPON1_BOVIN	Spondin-1
STIP1_BOVIN	Stress-induced-phosphoprotein 1
SUMO2_BOVIN	Small ubiquitin-related modifier 2
TBA1B_BOVIN	Tubulin alpha-1B chain
TBB2B_BOVIN	Tubulin beta-2B chain
TBB4A_BOVIN	Tubulin beta-4A chain
TCPA_BOVIN	T-complex protein 1 subunit alpha
TCPG_BOVIN	T-complex protein 1 subunit gamma
THRB_BOVIN	Prothrombin
TPM2_BOVIN	Tropomyosin beta chain
TRAP1_BOVIN	Heat shock protein 75 kDa, mitochondrial
TRY1_BOVIN	Cationic trypsin
TSP1_BOVIN	Thrombospondin-1
TTHY_BOVIN	Transthyretin
VDAC1_BOVIN	Voltage-dependent anion-selective channel protein 1
VDAC2_BOVIN	Voltage-dependent anion-selective channel protein 2
VIME_BOVIN	Vimentin
YBOX1_BOVIN	Nuclease-sensitive element-binding protein 1

Table S2: List of proteins recovered from PSS-coated AgNPs after 24 h incubation with NIH-3T3 medium

**Abbreviation**	**Name**
1433E_BOVIN	14-3-3 protein epsilon
A1AT_BOVIN	Alpha-1-antiproteinase
A1BG_BOVIN	Alpha-1B-glycoprotein
A2AP_BOVIN	Alpha-2-antiplasmin
A2MG_BOVIN	Alpha-2-macroglobulin
ACTB_BOVIN	Actin, cytoplasmic 1
ACTG_BOVIN	Actin, cytoplasmic 2
ALBU_BOVIN	Serum albumin
ALDOB_BOVIN	Fructose-bisphosphate aldolase B
ANXA2_BOVIN	Annexin A2

APOA1_BOVIN	Apolipoprotein A-I
APOE_BOVIN	Apolipoprotein E
ARF1_BOVIN	ADP-ribosylation factor 1
ATPA_BOVIN	ATP synthase subunit alpha, mitochondrial
ATPB_BOVIN	ATP synthase subunit beta, mitochondrial
BIP_BOVIN	Endoplasmic reticulum chaperone BiP
C1QA_BOVIN	Complement C1q subcomponent subunit A
C1QB_BOVIN	Complement C1q subcomponent subunit B
CFAB_BOVIN	Complement factor B
CFAH_BOVIN	Complement factor H
CH10_BOVIN	10 kDa heat shock protein, mitochondrial
CH60_BOVIN	60 kDa heat shock protein, mitochondrial
CNN3_BOVIN	Calponin-3
CO1A1_BOVIN	Collagen alpha-1(I) chain
CO3_BOVIN	Complement C3
CO4_BOVIN	Complement C4 (Fragments)
DDAH2_BOVIN	N(G),N(G)-dimethylarginine dimethylaminohydrolase 2
DHSO_BOVIN	Sorbitol dehydrogenase
EF1A1_BOVIN	Elongation factor 1-alpha 1
EF1D_BOVIN	Elongation factor 1-delta
EF2_BOVIN	Elongation factor 2
ENOA_BOVIN	Alpha-enolase
ENPL_BOVIN	Endoplasmin
EZRI_BOVIN	Ezrin
FA5_BOVIN	Coagulation factor V
FIBA_BOVIN	Fibrinogen alpha chain
FIBB_BOVIN	Fibrinogen beta chain
FIBG_BOVIN	Fibrinogen gamma-B chain
FINC_BOVIN	Fibronectin
G3P_BOVIN	Glyceraldehyde-3-phosphate dehydrogenase
GELS_BOVIN	Gelsolin
GLO2_BOVIN	Hydroxyacylglutathione hydrolase, mitochondrial
GSTP1_BOVIN	Glutathione S-transferase P
H11_BOVIN	Histone H1.1
H2A1_BOVIN	Histone H2A type 1
H2A2C_BOVIN	Histone H2A type 2-C
H2AV_BOVIN	Histone H2A.V
H2B1K_BOVIN	Histone H2B type 1-K
H4_BOVIN	Histone H4
HBA_BOVIN	Hemoglobin subunit alpha

HBBF_BOVIN	Hemoglobin fetal subunit beta
HEBP1_BOVIN	Heme-binding protein 1
HGFL_BOVIN	Hepatocyte growth factor-like protein
HS71A_BOVIN	Heat shock 70 kDa protein 1A
HS90A_BOVIN	Heat shock protein HSP 90-alpha
HS90B_BOVIN	Heat shock protein HSP 90-beta
HSP7C_BOVIN	Heat shock cognate 71 kDa protein
IPSP_BOVIN	Plasma serine protease inhibitor
ITIH1_BOVIN	Inter-alpha-trypsin inhibitor heavy chain H1
ITIH3_BOVIN	Inter-alpha-trypsin inhibitor heavy chain H3
ITIH4_BOVIN	Inter-alpha-trypsin inhibitor heavy chain H4
K2C75_BOVIN	Keratin, type II cytoskeletal 75
KNG1_BOVIN	Kininogen-1
LASP1_BOVIN	LIM and SH3 domain protein 1
LDHA_BOVIN	L-lactate dehydrogenase A chain
LDHB_BOVIN	L-lactate dehydrogenase B chain
MYL6_BOVIN	Myosin light polypeptide 6
MYL9_BOVIN	Myosin regulatory light polypeptide 9
NPM_BOVIN	Nucleophosmin
PDIA1_BOVIN	Protein disulfide-isomerase
PDIA3_BOVIN	Protein disulfide-isomerase A3
PEBP1_BOVIN	Phosphatidylethanolamine-binding protein 1
PEDF_BOVIN	Pigment epithelium-derived factor
PGK1_BOVIN	Phosphoglycerate kinase 1
PLMN_BOVIN	Plasminogen
PRDX1_BOVIN	Peroxiredoxin-1
RACK1_BOVIN	Receptor of activated protein C kinase 1
RGN_BOVIN	Regucalcin
RL11_BOVIN	60S ribosomal protein L11
RL14_BOVIN	60S ribosomal protein L14
ROA1_BOVIN	Heterogeneous nuclear ribonucleoprotein A1
RS13_BOVIN	40S ribosomal protein S13
RS24_BOVIN	40S ribosomal protein S24
RS27A_BOVIN	Ubiquitin-40S ribosomal protein S27a
RS28_BOVIN	40S ribosomal protein S28
RS4_BOVIN	40S ribosomal protein S4
RSSA_BOVIN	40S ribosomal protein SA
SAHH_BOVIN	Adenosylhomocysteinase
STMN1_BOVIN	Stathmin
SUMO2_BOVIN	Small ubiquitin-related modifier 2

TAGL_BOVIN	Transgelin
TAGL2_BOVIN	Transgelin-2
TBA1B_BOVIN	Tubulin alpha-1B chain
TBB2B_BOVIN	Tubulin beta-2B chain
TBB5_BOVIN	Tubulin beta-5 chain
TCPA_BOVIN	T-complex protein 1 subunit alpha
TETN_BOVIN	Tetranectin
THRB_BOVIN	Prothrombin
THYG_BOVIN	Thyroglobulin
TPM1_BOVIN	Tropomyosin alpha-1 chain
TPM2_BOVIN	Tropomyosin beta chain
TRFE_BOVIN	Serotransferrin
TRY1_BOVIN	Cationic trypsin
TSP1_BOVIN	Thrombospondin-1
VDAC1_BOVIN	Voltage-dependent anion-selective channel protein 1
VIME_BOVIN	Vimentin

Table S3: List of proteins recovered from PAH-coated AgNPs after 24 h incubation with NIH-3T3 medium

Abbreviation	Name
1433B_BOVIN	14-3-3 protein beta/alpha
1433E_BOVIN	14-3-3 protein epsilon
A1AT_BOVIN	Alpha-1-antiproteinase
A2MG_BOVIN	Alpha-2-macroglobulin
ACTA_BOVIN	Actin, aortic smooth muscle
ACTB_BOVIN	Actin, cytoplasmic 1
AINX_BOVIN	Alpha-internexin
ALBU_BOVIN	Serum albumin
ANXA2_BOVIN	Annexin A2
APOA1_BOVIN	Apolipoprotein A-I
APOE_BOVIN	Apolipoprotein E
ARF1_BOVIN	ADP-ribosylation factor 1
ATPA_BOVIN	ATP synthase subunit alpha, mitochondrial
BIP_BOVIN	Endoplasmic reticulum chaperone BiP
BLVRB_BOVIN	Flavin reductase (NADPH)
C1QA_BOVIN	Complement C1q subcomponent subunit A
C1QB_BOVIN	Complement C1q subcomponent subunit B
CALM_BOVIN	Calmodulin

CFAB_BOVIN	Complement factor B
CFAH_BOVIN	Complement factor H
CH10_BOVIN	10 kDa heat shock protein, mitochondrial
CH60_BOVIN	60 kDa heat shock protein, mitochondrial
CLUS_BOVIN	Clusterin
CO1A1_BOVIN	Collagen alpha-1(I) chain
CO2A1_BOVIN	Collagen alpha-1(II) chain
CO3_BOVIN	Complement C3
CO3A1_BOVIN	Collagen alpha-1(III) chain
CO4_BOVIN	Complement C4 (Fragments)
CRIP2_BOVIN	Cysteine-rich protein 2
CSRP1_BOVIN	Cysteine and glycine-rich protein 1
EF1A1_BOVIN	Elongation factor 1-alpha 1
EF1D_BOVIN	Elongation factor 1-delta
EF2_BOVIN	Elongation factor 2
EGFLA_BOVIN	Pikachurin
ENOA_BOVIN	Alpha-enolase
ENPL_BOVIN	Endoplasmin
EZRI_BOVIN	Ezrin
FETUA_BOVIN	Alpha-2-HS-glycoprotein
FETUB_BOVIN	Fetuin-B
FIBA_BOVIN	Fibrinogen alpha chain
FIBB_BOVIN	Fibrinogen beta chain
FINC_BOVIN	Fibronectin
G3P_BOVIN	Glyceraldehyde-3-phosphate dehydrogenase
GABT_BOVIN	4-aminobutyrate aminotransferase, mitochondrial
GELS_BOVIN	Gelsolin
GPC1_BOVIN	Glypican-1
GPX1_BOVIN	Glutathione peroxidase 1
H11_BOVIN	Histone H1.1
H2A1_BOVIN	Histone H2A type 1
H2A2C_BOVIN	Histone H2A type 2-C
H2B1K_BOVIN	Histone H2B type 1-K
H4_BOVIN	Histone H4
HBA_BOVIN	Hemoglobin subunit alpha
HBB_BOVIN	Hemoglobin subunit beta
HBBF_BOVIN	Hemoglobin fetal subunit beta
HMGB1_BOVIN	High mobility group protein B1
HMGB2_BOVIN	High mobility group protein B2
HS90A_BOVIN	Heat shock protein HSP 90-alpha

HS90B_BOVIN	Heat shock protein HSP 90-beta
HSP72_BOVIN	Heat shock-related 70 kDa protein 2
HSP7C_BOVIN	Heat shock cognate 71 kDa protein
IF4A1_BOVIN	Eukaryotic initiation factor 4A-I
ITIH3_BOVIN	Inter-alpha-trypsin inhibitor heavy chain H3
ITIH4_BOVIN	Inter-alpha-trypsin inhibitor heavy chain H4
K1C14_BOVIN	Keratin, type I cytoskeletal 14 (Fragment)
K2C5_BOVIN	Keratin, type II cytoskeletal 5
K2C75_BOVIN	Keratin, type II cytoskeletal 75
KNG1_BOVIN	Kininogen-1
LASP1_BOVIN	LIM and SH3 domain protein 1
LDHB_BOVIN	L-lactate dehydrogenase B chain
LECT2_BOVIN	Leukocyte cell-derived chemotaxin-2
MA2B1_BOVIN	Lysosomal alpha-mannosidase
MDHC_BOVIN	Malate dehydrogenase, cytoplasmic
MGP_BOVIN	Matrix Gla protein
MIF_BOVIN	Macrophage migration inhibitory factor
MYH10_BOVIN	Myosin-10
MYL9_BOVIN	Myosin regulatory light polypeptide 9
NDKA1_BOVIN	Nucleoside diphosphate kinase A 1
NP1L4_BOVIN	Nucleosome assembly protein 1-like 4
NRX1A_BOVIN	Neurexin-1
PARK7_BOVIN	Protein/nucleic acid deglycase DJ-1
PDIA1_BOVIN	Protein disulfide-isomerase
PEBP1_BOVIN	Phosphatidylethanolamine-binding protein 1
PGK1_BOVIN	Phosphoglycerate kinase 1
PGRP1_BOVIN	Peptidoglycan recognition protein 1
PLCB1_BOVIN	1-phosphatidylinositol 4,5-bisphosphate phosphodiesterase beta-1
PLMN_BOVIN	Plasminogen
PPBT_BOVIN	Alkaline phosphatase, tissue-nonspecific isozyme
PPIB_BOVIN	Peptidyl-prolyl cis-trans isomerase B
PRDX1_BOVIN	Peroxiredoxin-1
PROC_BOVIN	Vitamin K-dependent protein C (Fragment)
PROS_BOVIN	Vitamin K-dependent protein S
PSB5_BOVIN	Proteasome subunit beta type-5
PSB6_BOVIN	Proteasome subunit beta type-6
RACK1_BOVIN	Receptor of activated protein C kinase 1
RGN_BOVIN	Regucalcin
RL12_BOVIN	60S ribosomal protein L12
RL14_BOVIN	60S ribosomal protein L14

RL27_BOVIN	60S ribosomal protein L27
RLA0_BOVIN	60S acidic ribosomal protein P0
RLA2_BOVIN	60S acidic ribosomal protein P2
RN112_BOVIN	RING finger protein 112
ROA1_BOVIN	Heterogeneous nuclear ribonucleoprotein A1
RS13_BOVIN	40S ribosomal protein S13
RS26_BOVIN	40S ribosomal protein S26
RS28_BOVIN	40S ribosomal protein S28
RS4_BOVIN	40S ribosomal protein S4
SAHH_BOVIN	Adenosylhomocysteinase
SPP24_BOVIN	Secreted phosphoprotein 24
STIP1_BOVIN	Stress-induced-phosphoprotein 1
STMN1_BOVIN	Stathmin
SUMO2_BOVIN	Small ubiquitin-related modifier 2
TAGL2_BOVIN	Transgelin-2
TBA1B_BOVIN	Tubulin alpha-1B chain
TBB2B_BOVIN	Tubulin beta-2B chain
TBB4B_BOVIN	Tubulin beta-4B chain
TCPA_BOVIN	T-complex protein 1 subunit alpha
TEDC2_BOVIN	Tubulin epsilon and delta complex protein 2
THRB_BOVIN	Prothrombin
TPIS_BOVIN	Triosephosphate isomerase
TPM1_BOVIN	Tropomyosin alpha-1 chain
TRAP1_BOVIN	Heat shock protein 75 kDa, mitochondrial
TRY1_BOVIN	Cationic trypsin
TSP1_BOVIN	Thrombospondin-1
TSP4_BOVIN	Thrombospondin-4
TTC36_BOVIN	Tetratricopeptide repeat protein 36
VASP_BOVIN	Vasodilator-stimulated phosphoprotein
VIME_BOVIN	Vimentin

Table S4: List of proteins recovered from PDDA-coated AgNPs after 24 h incubation with NIH-3T3 medium

**Abbreviation**	**Name**
1433B_BOVIN	14-3-3 protein beta/alpha
A1AT_BOVIN	Alpha-1-antiproteinase
A1BG_BOVIN	Alpha-1B-glycoprotein
A2AP_BOVIN	Alpha-2-antiplasmin
A2MG_BOVIN	Alpha-2-macroglobulin
ACTB_BOVIN	Actin, cytoplasmic 1

ALBU_BOVIN	Serum albumin
ANGL4_BOVIN	Angiopoietin-related protein 4
ANT3_BOVIN	Antithrombin-III
ANXA2_BOVIN	Annexin A2
APOA1_BOVIN	Apolipoprotein A-I
APOE_BOVIN	Apolipoprotein E
ATPB_BOVIN	ATP synthase subunit beta, mitochondrial
BIP_BOVIN	Endoplasmic reticulum chaperone BiP
BLVRB_BOVIN	Flavin reductase (NADPH)
C1QA_BOVIN	Complement C1q subcomponent subunit A
CALR_BOVIN	Calreticulin
CATA_BOVIN	Catalase
CFAB_BOVIN	Complement factor B
CFAH_BOVIN	Complement factor H
CH10_BOVIN	10 kDa heat shock protein, mitochondrial
CH60_BOVIN	60 kDa heat shock protein, mitochondrial
CLCN7_BOVIN	H(+)/Cl(-) exchange transporter 7
CO1A1_BOVIN	Collagen alpha-1(I) chain
CO2A1_BOVIN	Collagen alpha-1(II) chain
CO3_BOVIN	Complement C3
CO4_BOVIN	Complement C4 (Fragments)
CO9_BOVIN	Complement component C9
CSPG2_BOVIN	Versican core protein
CTHL1_BOVIN	Cathelicidin-1
DYR_BOVIN	Dihydrofolate reductase
EF1A1_BOVIN	Elongation factor 1-alpha 1
EF2_BOVIN	Elongation factor 2
EGFLA_BOVIN	Pikachurin
ENOA_BOVIN	Alpha-enolase
ENPL_BOVIN	Endoplasmin
EZRI_BOVIN	Ezrin
F13A_BOVIN	Coagulation factor XIII A chain (Fragment)
FA5_BOVIN	Coagulation factor V
FETA_BOVIN	Alpha-fetoprotein
FETUB_BOVIN	Fetuin-B
FIBA_BOVIN	Fibrinogen alpha chain
FIBB_BOVIN	Fibrinogen beta chain
FIBG_BOVIN	Fibrinogen gamma-B chain
FINC_BOVIN	Fibronectin
GELS_BOVIN	Gelsolin

GPC1_BOVIN	Glypican-1
GPX1_BOVIN	Glutathione peroxidase 1
GRP75_BOVIN	Stress-70 protein, mitochondrial
H2A1_BOVIN	Histone H2A type 1
H2A2C_BOVIN	Histone H2A type 2-C
H2AV_BOVIN	Histone H2A.V
H2B1K_BOVIN	Histone H2B type 1-K
H31_BOVIN	Histone H3.1
H4_BOVIN	Histone H4
HBA_BOVIN	Hemoglobin subunit alpha
HBB_BOVIN	Hemoglobin subunit beta
HBBF_BOVIN	Hemoglobin fetal subunit beta
HEBP1_BOVIN	Heme-binding protein 1
HMGB2_BOVIN	High mobility group protein B2
HS71A_BOVIN	Heat shock 70 kDa protein 1A
HS90A_BOVIN	Heat shock protein HSP 90-alpha
HSP7C_BOVIN	Heat shock cognate 71 kDa protein
IBP5_BOVIN	Insulin-like growth factor-binding protein 5
IPSP_BOVIN	Plasma serine protease inhibitor
ITIH1_BOVIN	Inter-alpha-trypsin inhibitor heavy chain H1
ITIH3_BOVIN	Inter-alpha-trypsin inhibitor heavy chain H3
ITIH4_BOVIN	Inter-alpha-trypsin inhibitor heavy chain H4
K1C17_BOVIN	Keratin, type I cytoskeletal 17
K2C5_BOVIN	Keratin, type II cytoskeletal 5
KNG1_BOVIN	Kininogen-1
LASP1_BOVIN	LIM and SH3 domain protein 1
LDHB_BOVIN	L-lactate dehydrogenase B chain
LECT2_BOVIN	Leukocyte cell-derived chemotaxin-2
LUM_BOVIN	Lumican
MYH10_BOVIN	Myosin-10
MYL6_BOVIN	Myosin light polypeptide 6
MYL9_BOVIN	Myosin regulatory light polypeptide 9
NDKA1_BOVIN	Nucleoside diphosphate kinase A 1
PDIA1_BOVIN	Protein disulfide-isomerase
PEDF_BOVIN	Pigment epithelium-derived factor
PERI_BOVIN	Peripherin
PGCA_BOVIN	Aggrecan core protein
PGRP1_BOVIN	Peptidoglycan recognition protein 1
PGS1_BOVIN	Biglycan
PGS2_BOVIN	Decorin

PLMN_BOVIN	Plasminogen
PPIA_BOVIN	Peptidyl-prolyl cis-trans isomerase A
PRDX1_BOVIN	Peroxiredoxin-1
RGN_BOVIN	Regucalcin
RL14_BOVIN	60S ribosomal protein L14
RL23A_BOVIN	60S ribosomal protein L23a
RLA0_BOVIN	60S acidic ribosomal protein P0
RLA2_BOVIN	60S acidic ribosomal protein P2
ROA1_BOVIN	Heterogeneous nuclear ribonucleoprotein A1
ROA2_BOVIN	Heterogeneous nuclear ribonucleoproteins A2/B1
RS27A_BOVIN	Ubiquitin-40S ribosomal protein S27a
RS28_BOVIN	40S ribosomal protein S28
SHQ1_BOVIN	Protein SHQ1 homolog
SPP24_BOVIN	Secreted phosphoprotein 24
STIP1_BOVIN	Stress-induced-phosphoprotein 1
TAGL2_BOVIN	Transgelin-2
TBA1B_BOVIN	Tubulin alpha-1B chain
TBA4A_BOVIN	Tubulin alpha-4A chain
TBB2B_BOVIN	Tubulin beta-2B chain
TBB5_BOVIN	Tubulin beta-5 chain
THOC4_BOVIN	THO complex subunit 4
THRB_BOVIN	Prothrombin
THYG_BOVIN	Thyroglobulin
TPM1_BOVIN	Tropomyosin alpha-1 chain
TPM2_BOVIN	Tropomyosin beta chain
TRAP1_BOVIN	Heat shock protein 75 kDa, mitochondrial
TRFE_BOVIN	Serotransferrin
TRY1_BOVIN	Cationic trypsin
TSP1_BOVIN	Thrombospondin-1
TSP4_BOVIN	Thrombospondin-4
TTHY_BOVIN	Transthyretin
UBA1_BOVIN	Ubiquitin-like modifier-activating enzyme 1
VDAC1_BOVIN	Voltage-dependent anion-selective channel protein 1
VIME_BOVIN	Vimentin

Table S5: List of proteins recovered from citrate-coated AgNPs after 24 h incubation with MCF7/Caco2 medium

**Abbreviation**	**Name**
1433T_BOVIN	14-3-3 protein theta
1433Z_BOVIN	14-3-3 protein zeta/delta

A1AT_BOVIN	Alpha-1-antiproteinase
A1BG_BOVIN	Alpha-1B-glycoprotein
A2AP_BOVIN	Alpha-2-antiplasmin
A2MG_BOVIN	Alpha-2-macroglobulin
ABHEB_BOVIN	Protein ABHD14B
ACMSD_BOVIN	2-amino-3-carboxymuconate-6-semialdehyde decarboxylase
ACTB_BOVIN	Actin, cytoplasmic 1
ADIPO_BOVIN	Adiponectin
AK1A1_BOVIN	Alcohol dehydrogenase [NADP(+)]
AL8A1_BOVIN	Aldehyde dehydrogenase family 8 member A1
AL9A1_BOVIN	4-trimethylaminobutyraldehyde dehydrogenase
ALBU_BOVIN	Serum albumin
ALDH2_BOVIN	Aldehyde dehydrogenase, mitochondrial
ALDOB_BOVIN	Fructose-bisphosphate aldolase B
ALDR_BOVIN	Aldose reductase
AMBP_BOVIN	Protein AMBP
ANGL4_BOVIN	Angiopoietin-related protein 4
ANT3_BOVIN	Antithrombin-III
ANXA1_BOVIN	Annexin A1
ANXA5_BOVIN	Annexin A5
AP1M1_BOVIN	AP-1 complex subunit mu-1
APMAP_BOVIN	Adipocyte plasma membrane-associated protein
APOA1_BOVIN	Apolipoprotein A-I
APOA4_BOVIN	Apolipoprotein A-IV
APOE_BOVIN	Apolipoprotein E
ARC1B_BOVIN	Actin-related protein 2/3 complex subunit 1B
ARF1_BOVIN	ADP-ribosylation factor 1
ARF4_BOVIN	ADP-ribosylation factor 4
ARLY_BOVIN	Argininosuccinate lyase
ARP3_BOVIN	Actin-related protein 3
ASSY_BOVIN	Argininosuccinate synthase
B4GA1_BOVIN	Beta-1,4-glucuronyltransferase 1
B4GT1_BOVIN	Beta-1,4-galactosyltransferase 1
BGH3_BOVIN	Transforming growth factor-beta-induced protein ig-h3
BHMT1_BOVIN	Betaine--homocysteine S-methyltransferase 1
BIP_BOVIN	Endoplasmic reticulum chaperone BiP
BLVRB_BOVIN	Flavin reductase (NADPH)
BT3L4_BOVIN	Transcription factor BTF3 homolog 4
C1QA_BOVIN	Complement C1q subcomponent subunit A
C1QB_BOVIN	Complement C1q subcomponent subunit B

CAB39_BOVIN	Calcium-binding protein 39
CAH3_BOVIN	Carbonic anhydrase 3
CAN1_BOVIN	Calpain-1 catalytic subunit
CAN2_BOVIN	Calpain-2 catalytic subunit
CAPG_BOVIN	Macrophage-capping protein
CASB_BOVIN	Beta-casein
CATA_BOVIN	Catalase
CATB_BOVIN	Cathepsin B
CATC_BOVIN	Dipeptidyl peptidase 1
CATL1_BOVIN	Cathepsin L1
CBPB2_BOVIN	Carboxypeptidase B2
CBPN_BOVIN	Carboxypeptidase N catalytic chain
CBR1_BOVIN	Carbonyl reductase [NADPH] 1
CD14_BOVIN	Monocyte differentiation antigen CD14
CFAB_BOVIN	Complement factor B
CFAD_BOVIN	Complement factor D
CFAH_BOVIN	Complement factor H
CHAD_BOVIN	Chondroadherin
CL43_BOVIN	Collectin-43
CLIC1_BOVIN	Chloride intracellular channel protein 1
CLUS_BOVIN	Clusterin
CN37_BOVIN	2',3'-cyclic-nucleotide 3'-phosphodiesterase
CO1A1_BOVIN	Collagen alpha-1(I) chain
CO1A2_BOVIN	Collagen alpha-2(I) chain
CO2_BOVIN	Complement C2
CO2A1_BOVIN	Collagen alpha-1(II) chain
CO3_BOVIN	Complement C3
CO4_BOVIN	Complement C4 (Fragments)
CO7_BOVIN	Complement component C7
CO9_BOVIN	Complement component C9
COMP_BOVIN	Cartilage oligomeric matrix protein
CONG_BOVIN	Conglutinin
COR1A_BOVIN	Coronin-1A
COTL1_BOVIN	Coactosin-like protein
CPNS1_BOVIN	Calpain small subunit 1
CRIP2_BOVIN	Cysteine-rich protein 2
CSRP1_BOVIN	Cysteine and glycine-rich protein 1
CTHL1_BOVIN	Cathelicidin-1
CTHL4_BOVIN	Cathelicidin-4
CYBP_BOVIN	Calcyclin-binding protein

DDAH2_BOVIN	N(G),N(G)-dimethylarginine dimethylaminohydrolase 2
DDBX_BOVIN	Dihydrodiol dehydrogenase 3
DHSO_BOVIN	Sorbitol dehydrogenase
DNJA2_BOVIN	DnaJ homolog subfamily A member 2
DPYD_BOVIN	Dihydropyrimidine dehydrogenase [NADP(+)]
DTD1_BOVIN	D-aminoacyl-tRNA deacylase 1
DYR_BOVIN	Dihydrofolate reductase
EF1A1_BOVIN	Elongation factor 1-alpha 1
EF1D_BOVIN	Elongation factor 1-delta
EF1G_BOVIN	Elongation factor 1-gamma
EF2_BOVIN	Elongation factor 2
ENOA_BOVIN	Alpha-enolase
ENPL_BOVIN	Endoplasmin
EZRI_BOVIN	Ezrin
F12AI_BOVIN	Factor XIIa inhibitor
F13A_BOVIN	Coagulation factor XIII A chain (Fragment)
F16P1_BOVIN	Fructose-1,6-bisphosphatase 1
FA10_BOVIN	Coagulation factor X
FA11_BOVIN	Coagulation factor XI
FA12_BOVIN	Coagulation factor XII
FA5_BOVIN	Coagulation factor V
FAAA_BOVIN	Fumarylacetoacetase
FCN2_BOVIN	Ficolin-2
FETA_BOVIN	Alpha-fetoprotein
FETUA_BOVIN	Alpha-2-HS-glycoprotein
FETUB_BOVIN	Fetuin-B
FIBA_BOVIN	Fibrinogen alpha chain
FIBB_BOVIN	Fibrinogen beta chain
FIBG_BOVIN	Fibrinogen gamma-B chain
FINC_BOVIN	Fibronectin
FKB1A_BOVIN	Peptidyl-prolyl cis-trans isomerase FKBP1A
G3P_BOVIN	Glyceraldehyde-3-phosphate dehydrogenase
GALK1_BOVIN	Galactokinase
GALM_BOVIN	Aldose 1-epimerase
GDIB_BOVIN	Rab GDP dissociation inhibitor beta
GELS_BOVIN	Gelsolin
GGH_BOVIN	Gamma-glutamyl hydrolase
GLO2_BOVIN	Hydroxyacylglutathione hydrolase, mitochondrial
GSHB_BOVIN	Glutathione synthetase
H2A1_BOVIN	Histone H2A type 1

H2A2C_BOVIN	Histone H2A type 2-C
H2B1K_BOVIN	Histone H2B type 1-K
H4_BOVIN	Histone H4
HA1B_BOVIN	BOLA class I histocompatibility antigen, alpha chain BL3-7
HABP2_BOVIN	Hyaluronan-binding protein 2
HBA_BOVIN	Hemoglobin subunit alpha
HBB_BOVIN	Hemoglobin subunit beta
HBBF_BOVIN	Hemoglobin fetal subunit beta
HEBP1_BOVIN	Heme-binding protein 1
HEM2_BOVIN	Delta-aminolevulinic acid dehydratase
HEM3_BOVIN	Porphobilinogen deaminase
HEMO_BOVIN	Hemopexin
HMGB2_BOVIN	High mobility group protein B2
HMGN2_BOVIN	Non-histone chromosomal protein HMG-17
HNRPK_BOVIN	Heterogeneous nuclear ribonucleoprotein K
HP20_BOVIN	Protein HP-20 homolog
HS71A_BOVIN	Heat shock 70 kDa protein 1A
HS90A_BOVIN	Heat shock protein HSP 90-alpha
HS90B_BOVIN	Heat shock protein HSP 90-beta
HSP72_BOVIN	Heat shock-related 70 kDa protein 2
HSP7C_BOVIN	Heat shock cognate 71 kDa protein
HSPB1_BOVIN	Heat shock protein beta-1
HTRA1_BOVIN	Serine protease HTRA1
IBP2_BOVIN	Insulin-like growth factor-binding protein 2
IBP3_BOVIN	Insulin-like growth factor-binding protein 3
IBP4_BOVIN	Insulin-like growth factor-binding protein 4
IBP5_BOVIN	Insulin-like growth factor-binding protein 5
IBP6_BOVIN	Insulin-like growth factor-binding protein 6
IF2A_BOVIN	Eukaryotic translation initiation factor 2 subunit 1
IF4A1_BOVIN	Eukaryotic initiation factor 4A-I
IKIP_BOVIN	Inhibitor of nuclear factor kappa-B kinase-interacting protein
ILK_BOVIN	Integrin-linked protein kinase
IPSP_BOVIN	Plasma serine protease inhibitor
ITIH1_BOVIN	Inter-alpha-trypsin inhibitor heavy chain H1
ITIH3_BOVIN	Inter-alpha-trypsin inhibitor heavy chain H3
ITIH4_BOVIN	Inter-alpha-trypsin inhibitor heavy chain H4
ITIH5_BOVIN	Inter-alpha-trypsin inhibitor heavy chain H5
K1C10_BOVIN	Keratin, type I cytoskeletal 10
K1C17_BOVIN	Keratin, type I cytoskeletal 17
K2C5_BOVIN	Keratin, type II cytoskeletal 5

K2C7_BOVIN	Keratin, type II cytoskeletal 7
K2C79_BOVIN	Keratin, type II cytoskeletal 79
KAP0_BOVIN	cAMP-dependent protein kinase type I-alpha regulatory subunit
KCRB_BOVIN	Creatine kinase B-type
KCRM_BOVIN	Creatine kinase M-type
KLKB1_BOVIN	Plasma kallikrein
KNG1_BOVIN	Kininogen-1
LASP1_BOVIN	LIM and SH3 domain protein 1
LDHA_BOVIN	L-lactate dehydrogenase A chain
LDHB_BOVIN	L-lactate dehydrogenase B chain
LUM_BOVIN	Lumican
MAP4_BOVIN	Microtubule-associated protein 4
MDHC_BOVIN	Malate dehydrogenase, cytoplasmic
METK1_BOVIN	S-adenosylmethionine synthase isoform type-1
MGP_BOVIN	Matrix Gla protein
MIF_BOVIN	Macrophage migration inhibitory factor
MIME_BOVIN	Mimecan
MK01_BOVIN	Mitogen-activated protein kinase 1
MOES_BOVIN	Moesin
MTAP_BOVIN	S-methyl-5'-thioadenosine phosphorylase
MYH10_BOVIN	Myosin-10
MYL6_BOVIN	Myosin light polypeptide 6
MYL9_BOVIN	Myosin regulatory light polypeptide 9
MYOC_BOVIN	Myocilin
NADC_BOVIN	Nicotinate-nucleotide pyrophosphorylase [carboxylating]
NB5R3_BOVIN	NADH-cytochrome b5 reductase 3
NDKB_BOVIN	Nucleoside diphosphate kinase B
NP1L1_BOVIN	Nucleosome assembly protein 1-like 1
NP1L4_BOVIN	Nucleosome assembly protein 1-like 4
OLFL3_BOVIN	Olfactomedin-like protein 3
PBLD_BOVIN	Phenazine biosynthesis-like domain-containing protein
PDLI1_BOVIN	PDZ and LIM domain protein 1
PEBP1_BOVIN	Phosphatidylethanolamine-binding protein 1
PEDF_BOVIN	Pigment epithelium-derived factor
PERI_BOVIN	Peripherin
PGK1_BOVIN	Phosphoglycerate kinase 1
PGRP1_BOVIN	Peptidoglycan recognition protein 1
PHLD_BOVIN	Phosphatidylinositol-glycan-specific phospholipase D
PLMN_BOVIN	Plasminogen
PP1A_BOVIN	Serine/threonine-protein phosphatase PP1-alpha catalytic subunit

PP1R7_BOVIN	Protein phosphatase 1 regulatory subunit 7
PPBT_BOVIN	Alkaline phosphatase, tissue-nonspecific isozyme
PPIA_BOVIN	Peptidyl-prolyl cis-trans isomerase A
PPIB_BOVIN	Peptidyl-prolyl cis-trans isomerase B
PPWD1_BOVIN	Peptidylprolyl isomerase domain and WD repeat-containing protein 1
PRDX1_BOVIN	Peroxiredoxin-1
PRDX2_BOVIN	Peroxiredoxin-2
PRDX6_BOVIN	Peroxiredoxin-6
PROC_BOVIN	Vitamin K-dependent protein C (Fragment)
PROS_BOVIN	Vitamin K-dependent protein S
PRS7_BOVIN	26S proteasome regulatory subunit 7
PSA1_BOVIN	Proteasome subunit alpha type-1
PSA4_BOVIN	Proteasome subunit alpha type-4
PSA6_BOVIN	Proteasome subunit alpha type-6
PSA7_BOVIN	Proteasome subunit alpha type-7
PSB4_BOVIN	Proteasome subunit beta type-4
PSB5_BOVIN	Proteasome subunit beta type-5
PSB6_BOVIN	Proteasome subunit beta type-6
PSB7_BOVIN	Proteasome subunit beta type-7
PSB9_BOVIN	Proteasome subunit beta type-9
PTPRF_BOVIN	Receptor-type tyrosine-protein phosphatase F
PTX3_BOVIN	Pentraxin-related protein PTX3
RAB8A_BOVIN	Ras-related protein Rab-8A
RAC1_BOVIN	Ras-related C3 botulinum toxin substrate 1
RAN_BOVIN	GTP-binding nuclear protein Ran
RET1_BOVIN	Retinol-binding protein 1
RGN_BOVIN	Regucalcin
RHOA_BOVIN	Transforming protein RhoA
RLA2_BOVIN	60S acidic ribosomal protein P2
RS24_BOVIN	40S ribosomal protein S24
RS28_BOVIN	40S ribosomal protein S28
S10A8_BOVIN	Protein S100-A8
S10AC_BOVIN	Protein S100-A12
S14L2_BOVIN	SEC14-like protein 2
SAHH_BOVIN	Adenosylhomocysteinase
SAMP_BOVIN	Serum amyloid P-component
SBP1_BOVIN	Methanethiol oxidase
SEPT2_BOVIN	Septin-2
SEPT7_BOVIN	Septin-7
SERA_BOVIN	D-3-phosphoglycerate dehydrogenase

SERPH_BOVIN	Serpin H1
SFTPD_BOVIN	Pulmonary surfactant-associated protein D
SPA31_BOVIN	Serpin A3-1
SPON1_BOVIN	Spondin-1
SPP24_BOVIN	Secreted phosphoprotein 24
ST1A1_BOVIN	Sulfotransferase 1A1
STIP1_BOVIN	Stress-induced-phosphoprotein 1
STK10_BOVIN	Serine/threonine-protein kinase 10
SYTC_BOVIN	Threonine--tRNA ligase, cytoplasmic
TAGL_BOVIN	Transgelin
TAGL2_BOVIN	Transgelin-2
TBA1B_BOVIN	Tubulin alpha-1B chain
TBA1C_BOVIN	Tubulin alpha-1C chain
TBA4A_BOVIN	Tubulin alpha-4A chain
TBB2B_BOVIN	Tubulin beta-2B chain
TBB4A_BOVIN	Tubulin beta-4A chain
TBB4B_BOVIN	Tubulin beta-4B chain
TBB5_BOVIN	Tubulin beta-5 chain
TES_BOVIN	Testin
TETN_BOVIN	Tetranectin
THBG_BOVIN	Thyroxine-binding globulin
THRB_BOVIN	Prothrombin
TIMP2_BOVIN	Metalloproteinase inhibitor 2
TKT_BOVIN	Transketolase
TPIS_BOVIN	Triosephosphate isomerase
TPM2_BOVIN	Tropomyosin beta chain
TRFE_BOVIN	Serotransferrin
TRFL_BOVIN	Lactotransferrin
TRY1_BOVIN	Cationic trypsin
TSN_BOVIN	Translin
TSP1_BOVIN	Thrombospondin-1
TSP4_BOVIN	Thrombospondin-4
TTHY_BOVIN	Transthyretin
URP2_BOVIN	Fermitin family homolog 3
VASP_BOVIN	Vasodilator-stimulated phosphoprotein
VTDB_BOVIN	Vitamin D-binding protein
WDR1_BOVIN	WD repeat-containing protein 1
YBOX1_BOVIN	Nuclease-sensitive element-binding protein 1

Table S6: List of proteins recovered from PSS-coated AgNPs after 24 h incubation with MCF7/Caco2 medium

Abbreviation	Name
A1AG_BOVIN	Alpha-1-acid glycoprotein
A1AT_BOVIN	Alpha-1-antiproteinase
A1BG_BOVIN	Alpha-1B-glycoprotein
A2MG_BOVIN	Alpha-2-macroglobulin
ABHEB_BOVIN	Protein ABHD14B
ACLY_BOVIN	ATP-citrate synthase
ACTB_BOVIN	Actin, cytoplasmic 1
ACTG_BOVIN	Actin, cytoplasmic 2
ALBU_BOVIN	Serum albumin
ALDOB_BOVIN	Fructose-bisphosphate aldolase B
APOA1_BOVIN	Apolipoprotein A-I
APOA4_BOVIN	Apolipoprotein A-IV
APOE_BOVIN	Apolipoprotein E
ARF1_BOVIN	ADP-ribosylation factor 1
ARP2_BOVIN	Actin-related protein 2
ARPC4_BOVIN	Actin-related protein 2/3 complex subunit 4
BIP_BOVIN	Endoplasmic reticulum chaperone BiP
C1QA_BOVIN	Complement C1q subcomponent subunit A
C1QB_BOVIN	Complement C1q subcomponent subunit B
CAP1_BOVIN	Adenylyl cyclase-associated protein 1
CASB_BOVIN	Beta-casein
CATA_BOVIN	Catalase
CATC_BOVIN	Dipeptidyl peptidase 1
CBPB2_BOVIN	Carboxypeptidase B2
CDC42_BOVIN	Cell division control protein 42 homolog
CFAB_BOVIN	Complement factor B
CFAD_BOVIN	Complement factor D
CFAH_BOVIN	Complement factor H
CL43_BOVIN	Collectin-43
CLUS_BOVIN	Clusterin
CNTN1_BOVIN	Contactin-1
CO1A1_BOVIN	Collagen alpha-1(I) chain
CO3_BOVIN	Complement C3
CO4_BOVIN	Complement C4 (Fragments)
CO6_BOVIN	Complement component C6
CO7_BOVIN	Complement component C7
CO9_BOVIN	Complement component C9

COF1_BOVIN	Cofilin-1
COF2_BOVIN	Cofilin-2
COMP_BOVIN	Cartilage oligomeric matrix protein
COTL1_BOVIN	Coactosin-like protein
CPNS1_BOVIN	Calpain small subunit 1
CSN3_BOVIN	COP9 signalosome complex subunit 3
CSRP1_BOVIN	Cysteine and glycine-rich protein 1
CTHL1_BOVIN	Cathelicidin-1
CTHL4_BOVIN	Cathelicidin-4
DDAH2_BOVIN	N(G),N(G)-dimethylarginine dimethylaminohydrolase 2
DHSO_BOVIN	Sorbitol dehydrogenase
EF1A1_BOVIN	Elongation factor 1-alpha 1
EF1G_BOVIN	Elongation factor 1-gamma
ENOA_BOVIN	Alpha-enolase
ENPL_BOVIN	Endoplasmin
ESTD_BOVIN	S-formylglutathione hydrolase
F13A_BOVIN	Coagulation factor XIII A chain (Fragment)
F16P1_BOVIN	Fructose-1,6-bisphosphatase 1
FA12_BOVIN	Coagulation factor XII
FA5_BOVIN	Coagulation factor V
FAM3C_BOVIN	Protein FAM3C
FEN1_BOVIN	Flap endonuclease 1
FETA_BOVIN	Alpha-fetoprotein
FETUA_BOVIN	Alpha-2-HS-glycoprotein
FETUB_BOVIN	Fetuin-B
FIBA_BOVIN	Fibrinogen alpha chain
FIBB_BOVIN	Fibrinogen beta chain
FIBG_BOVIN	Fibrinogen gamma-B chain
FINC_BOVIN	Fibronectin
FKB1A_BOVIN	Peptidyl-prolyl cis-trans isomerase FKBP1A
G3P_BOVIN	Glyceraldehyde-3-phosphate dehydrogenase
GALK1_BOVIN	Galactokinase
GELS_BOVIN	Gelsolin
GGACT_BOVIN	Gamma-glutamylaminecyclotransferase
GGT7_BOVIN	Glutathione hydrolase 7
GLO2_BOVIN	Hydroxyacylglutathione hydrolase, mitochondrial
GPX1_BOVIN	Glutathione peroxidase 1
GSTP1_BOVIN	Glutathione S-transferase P
H2A1_BOVIN	Histone H2A type 1
H2A2C_BOVIN	Histone H2A type 2-C

H2B1K_BOVIN	Histone H2B type 1-K
H4_BOVIN	Histone H4
HBA_BOVIN	Hemoglobin subunit alpha
HBB_BOVIN	Hemoglobin subunit beta
HBBF_BOVIN	Hemoglobin fetal subunit beta
HEBP1_BOVIN	Heme-binding protein 1
HEM2_BOVIN	Delta-aminolevulinic acid dehydratase
HGFL_BOVIN	Hepatocyte growth factor-like protein
HP20_BOVIN	Protein HP-20 homolog
HS71L_BOVIN	Heat shock 70 kDa protein 1-like
HS90A_BOVIN	Heat shock protein HSP 90-alpha
HSP7C_BOVIN	Heat shock cognate 71 kDa protein
HSPB1_BOVIN	Heat shock protein beta-1
IBP2_BOVIN	Insulin-like growth factor-binding protein 2
IBP6_BOVIN	Insulin-like growth factor-binding protein 6
IGF2_BOVIN	Insulin-like growth factor II
IPSP_BOVIN	Plasma serine protease inhibitor
ITIH1_BOVIN	Inter-alpha-trypsin inhibitor heavy chain H1
ITIH3_BOVIN	Inter-alpha-trypsin inhibitor heavy chain H3
ITIH4_BOVIN	Inter-alpha-trypsin inhibitor heavy chain H4
K1C10_BOVIN	Keratin, type I cytoskeletal 10
K2C79_BOVIN	Keratin, type II cytoskeletal 79
KAD1_BOVIN	Adenylate kinase isoenzyme 1
KCRB_BOVIN	Creatine kinase B-type
KERA_BOVIN	Keratocan
KNG1_BOVIN	Kininogen-1
LASP1_BOVIN	LIM and SH3 domain protein 1
LDHA_BOVIN	L-lactate dehydrogenase A chain
LDHB_BOVIN	L-lactate dehydrogenase B chain
LYPA1_BOVIN	Acyl-protein thioesterase 1
MARE1_BOVIN	Microtubule-associated protein RP/EB family member 1
MDHC_BOVIN	Malate dehydrogenase, cytoplasmic
METK1_BOVIN	S-adenosylmethionine synthase isoform type-1
MGP_BOVIN	Matrix Gla protein
MIF_BOVIN	Macrophage migration inhibitory factor
MOES_BOVIN	Moesin
MPRI_BOVIN	Cation-independent mannose-6-phosphate receptor
MYH10_BOVIN	Myosin-10
MYL6_BOVIN	Myosin light polypeptide 6
OSTF1_BOVIN	Osteoclast-stimulating factor 1

PDIA1_BOVIN	Protein disulfide-isomerase
PDLI1_BOVIN	PDZ and LIM domain protein 1
PDLI3_BOVIN	PDZ and LIM domain protein 3
PEBP1_BOVIN	Phosphatidylethanolamine-binding protein 1
PEDF_BOVIN	Pigment epithelium-derived factor
PERI_BOVIN	Peripherin
PGRP1_BOVIN	Peptidoglycan recognition protein 1
PLMN_BOVIN	Plasminogen
PPIA_BOVIN	Peptidyl-prolyl cis-trans isomerase A
PRDX1_BOVIN	Peroxiredoxin-1
PRDX2_BOVIN	Peroxiredoxin-2
PRDX6_BOVIN	Peroxiredoxin-6
PROS_BOVIN	Vitamin K-dependent protein S
PSA6_BOVIN	Proteasome subunit alpha type-6
PSA7_BOVIN	Proteasome subunit alpha type-7
PSB1_BOVIN	Proteasome subunit beta type-1
PSB10_BOVIN	Proteasome subunit beta type-10
PSB2_BOVIN	Proteasome subunit beta type-2
PSB3_BOVIN	Proteasome subunit beta type-3
PSB4_BOVIN	Proteasome subunit beta type-4
PTGR1_BOVIN	Prostaglandin reductase 1
RAB7A_BOVIN	Ras-related protein Rab-7a
RAP1A_BOVIN	Ras-related protein Rap-1A
RGN_BOVIN	Regucalcin
RGS10_BOVIN	Regulator of G-protein signaling 10
RL14_BOVIN	60S ribosomal protein L14
RLA1_BOVIN	60S acidic ribosomal protein P1
RS18_BOVIN	40S ribosomal protein S18
RTEL1_BOVIN	Regulator of telomere elongation helicase 1
SAHH_BOVIN	Adenosylhomocysteinase
SEPT2_BOVIN	Septin-2
SERPH_BOVIN	Serpin H1
SNX3_BOVIN	Sorting nexin-3
SPP24_BOVIN	Secreted phosphoprotein 24
ST1E1_BOVIN	Estrogen sulfotransferase
STMN1_BOVIN	Stathmin
STX7_BOVIN	Syntaxin-7
SUMO2_BOVIN	Small ubiquitin-related modifier 2
TAGL_BOVIN	Transgelin
TAGL2_BOVIN	Transgelin-2

TBA1B_BOVIN	Tubulin alpha-1B chain
TBA4A_BOVIN	Tubulin alpha-4A chain
TERA_BOVIN	Transitional endoplasmic reticulum ATPase
TES_BOVIN	Testin
THRB_BOVIN	Prothrombin
THYG_BOVIN	Thyroglobulin
TKT_BOVIN	Transketolase
TOLIP_BOVIN	Toll-interacting protein
TPIS_BOVIN	Triosephosphate isomerase
TPM2_BOVIN	Tropomyosin beta chain
TRAP1_BOVIN	Heat shock protein 75 kDa, mitochondrial
TRFE_BOVIN	Serotransferrin
TRY1_BOVIN	Cationic trypsin
TSP1_BOVIN	Thrombospondin-1
TSP4_BOVIN	Thrombospondin-4
TTHY_BOVIN	Transthyretin
UB2V1_BOVIN	Ubiquitin-conjugating enzyme E2 variant 1
VASP_BOVIN	Vasodilator-stimulated phosphoprotein
VDAC1_BOVIN	Voltage-dependent anion-selective channel protein 1
VIME_BOVIN	Vimentin

Table S7: List of proteins recovered from PAH-coated AgNPs after 24 h incubation with MCF7/Caco2 medium

Abbreviation	Name
A1AT_BOVIN	Alpha-1-antiproteinase
A1BG_BOVIN	Alpha-1B-glycoprotein
A2AP_BOVIN	Alpha-2-antiplasmin
A2MG_BOVIN	Alpha-2-macroglobulin
ACTB_BOVIN	Actin, cytoplasmic 1
ALBU_BOVIN	Serum albumin
ANGL4_BOVIN	Angiopoietin-related protein 4
ANXA2_BOVIN	Annexin A2
AP4S1_BOVIN	AP-4 complex subunit sigma-1
APOA1_BOVIN	Apolipoprotein A-I
APOE_BOVIN	Apolipoprotein E
ARF1_BOVIN	ADP-ribosylation factor 1
ATPA_BOVIN	ATP synthase subunit alpha, mitochondrial
ATPB_BOVIN	ATP synthase subunit beta, mitochondrial
BIP_BOVIN	Endoplasmic reticulum chaperone BiP
CASA1_BOVIN	Alpha-S1-casein

CASB_BOVIN	Beta-casein
CFAB_BOVIN	Complement factor B
CFAH_BOVIN	Complement factor H
CH10_BOVIN	10 kDa heat shock protein, mitochondrial
CH60_BOVIN	60 kDa heat shock protein, mitochondrial
CLUS_BOVIN	Clusterin
CO1A1_BOVIN	Collagen alpha-1(I) chain
CO2_BOVIN	Complement C2
CO2A1_BOVIN	Collagen alpha-1(II) chain
CO3_BOVIN	Complement C3
CO3A1_BOVIN	Collagen alpha-1(III) chain
CO4_BOVIN	Complement C4 (Fragments)
CO9_BOVIN	Complement component C9
COAA1_BOVIN	Collagen alpha-1(X) chain
CSPG2_BOVIN	Versican core protein
EF1A1_BOVIN	Elongation factor 1-alpha 1
EF2_BOVIN	Elongation factor 2
EIF1A_BOVIN	Probable RNA-binding protein EIF1AD
ENOA_BOVIN	Alpha-enolase
ENPL_BOVIN	Endoplasmin
EZRI_BOVIN	Ezrin
F13A_BOVIN	Coagulation factor XIII A chain (Fragment)
FA5_BOVIN	Coagulation factor V
FETA_BOVIN	Alpha-fetoprotein
FETUA_BOVIN	Alpha-2-HS-glycoprotein
FETUB_BOVIN	Fetuin-B
FIBA_BOVIN	Fibrinogen alpha chain
FIBB_BOVIN	Fibrinogen beta chain
FIBG_BOVIN	Fibrinogen gamma-B chain
FINC_BOVIN	Fibronectin
GELS_BOVIN	Gelsolin
GPC1_BOVIN	Glypican-1
GSTP1_BOVIN	Glutathione S-transferase P
H11_BOVIN	Histone H1.1
H2A1_BOVIN	Histone H2A type 1
H2AV_BOVIN	Histone H2A.V
H2B1K_BOVIN	Histone H2B type 1-K
H4_BOVIN	Histone H4
HBA_BOVIN	Hemoglobin subunit alpha
HBB_BOVIN	Hemoglobin subunit beta

HBBF_BOVIN	Hemoglobin fetal subunit beta
HMGB2_BOVIN	High mobility group protein B2
HS71A_BOVIN	Heat shock 70 kDa protein 1A
HS71L_BOVIN	Heat shock 70 kDa protein 1-like
HS90A_BOVIN	Heat shock protein HSP 90-alpha
HS90B_BOVIN	Heat shock protein HSP 90-beta
HSP7C_BOVIN	Heat shock cognate 71 kDa protein
ITIH1_BOVIN	Inter-alpha-trypsin inhibitor heavy chain H1
ITIH3_BOVIN	Inter-alpha-trypsin inhibitor heavy chain H3
ITIH4_BOVIN	Inter-alpha-trypsin inhibitor heavy chain H4
K1C17_BOVIN	Keratin, type I cytoskeletal 17
K2C5_BOVIN	Keratin, type II cytoskeletal 5
KNG1_BOVIN	Kininogen-1
LDHB_BOVIN	L-lactate dehydrogenase B chain
LUM_BOVIN	Lumican
MA2B1_BOVIN	Lysosomal alpha-mannosidase
MGP_BOVIN	Matrix Gla protein
MIF_BOVIN	Macrophage migration inhibitory factor
MYH10_BOVIN	Myosin-10
MYL6_BOVIN	Myosin light polypeptide 6
NP1L4_BOVIN	Nucleosome assembly protein 1-like 4
NRX1A_BOVIN	Neurexin-1
OLM2B_BOVIN	Olfactomedin-like protein 2B
PDIA1_BOVIN	Protein disulfide-isomerase
PDIA4_BOVIN	Protein disulfide-isomerase A4
PERI_BOVIN	Peripherin
PGCA_BOVIN	Aggrecan core protein
PGRP1_BOVIN	Peptidoglycan recognition protein 1
PGS1_BOVIN	Biglycan
PGS2_BOVIN	Decorin
PLMN_BOVIN	Plasminogen
PRDX1_BOVIN	Peroxiredoxin-1
PROS_BOVIN	Vitamin K-dependent protein S
RAB1B_BOVIN	Ras-related protein Rab-1B
RGN_BOVIN	Regucalcin
RL32_BOVIN	60S ribosomal protein L32
RL9_BOVIN	60S ribosomal protein L9
RLA0_BOVIN	60S acidic ribosomal protein P0
RLA2_BOVIN	60S acidic ribosomal protein P2
RS25_BOVIN	40S ribosomal protein S25

RS8_BOVIN	40S ribosomal protein S8
RSSA_BOVIN	40S ribosomal protein SA
SPP24_BOVIN	Secreted phosphoprotein 24
SUMO2_BOVIN	Small ubiquitin-related modifier 2
TAGL2_BOVIN	Transgelin-2
TBA1B_BOVIN	Tubulin alpha-1B chain
TBB2B_BOVIN	Tubulin beta-2B chain
TCPG_BOVIN	T-complex protein 1 subunit gamma
TEDC2_BOVIN	Tubulin epsilon and delta complex protein 2
TETN_BOVIN	Tetranectin
THBG_BOVIN	Thyroxine-binding globulin
THIO_BOVIN	Thioredoxin
THRB_BOVIN	Prothrombin
THYG_BOVIN	Thyroglobulin
TPM1_BOVIN	Tropomyosin alpha-1 chain
TRFE_BOVIN	Serotransferrin
TRFL_BOVIN	Lactotransferrin
TSP1_BOVIN	Thrombospondin-1
TSP4_BOVIN	Thrombospondin-4
UBA1_BOVIN	Ubiquitin-like modifier-activating enzyme 1
VIME_BOVIN	Vimentin
YBOX1_BOVIN	Nuclease-sensitive element-binding protein 1

Table S8: List of proteins recovered from PDDA-coated AgNPs after 24 h incubation with MCF7/Caco2 medium

**Abbreviation**	**Name**
A16A1_BOVIN	Aldehyde dehydrogenase family 16 member A1
A1AT_BOVIN	Alpha-1-antiproteinase
A1BG_BOVIN	Alpha-1B-glycoprotein
A2MG_BOVIN	Alpha-2-macroglobulin
ACMSD_BOVIN	2-amino-3-carboxymuconate-6-semialdehyde decarboxylase
ACOC_BOVIN	Cytoplasmic aconitate hydratase
ACTA_BOVIN	Actin, aortic smooth muscle
ACTB_BOVIN	Actin, cytoplasmic 1
ALBU_BOVIN	Serum albumin
ALDOB_BOVIN	Fructose-bisphosphate aldolase B
ANT3_BOVIN	Antithrombin-III
ANXA2_BOVIN	Annexin A2
ANXA6_BOVIN	Annexin A6
AP1S2_BOVIN	AP-1 complex subunit sigma-2

APOA1_BOVIN	Apolipoprotein A-I
APOA4_BOVIN	Apolipoprotein A-IV
APOE_BOVIN	Apolipoprotein E
ARF1_BOVIN	ADP-ribosylation factor 1
ATPB_BOVIN	ATP synthase subunit beta, mitochondrial
BIP_BOVIN	Endoplasmic reticulum chaperone BiP
C1QA_BOVIN	Complement C1q subcomponent subunit A
C1QB_BOVIN	Complement C1q subcomponent subunit B
C1S_BOVIN	Complement C1s subcomponent
CALM_BOVIN	Calmodulin
CBPN_BOVIN	Carboxypeptidase N catalytic chain
CFAB_BOVIN	Complement factor B
CFAH_BOVIN	Complement factor H
CH60_BOVIN	60 kDa heat shock protein, mitochondrial
CLUS_BOVIN	Clusterin
CNN3_BOVIN	Calponin-3
CO1A1_BOVIN	Collagen alpha-1(I) chain
CO3_BOVIN	Complement C3
CO3A1_BOVIN	Collagen alpha-1(III) chain
CO4_BOVIN	Complement C4 (Fragments)
CO9_BOVIN	Complement component C9
COF1_BOVIN	Cofilin-1
COTL1_BOVIN	Coactosin-like protein
CSRP1_BOVIN	Cysteine and glycine-rich protein 1
DDAH2_BOVIN	N(G),N(G)-dimethylarginine dimethylaminohydrolase 2
EF1A1_BOVIN	Elongation factor 1-alpha 1
EF2_BOVIN	Elongation factor 2
ENOA_BOVIN	Alpha-enolase
ENPL_BOVIN	Endoplasmin
F13A_BOVIN	Coagulation factor XIII A chain (Fragment)
FA10_BOVIN	Coagulation factor X
FA5_BOVIN	Coagulation factor V
FETA_BOVIN	Alpha-fetoprotein
FETUA_BOVIN	Alpha-2-HS-glycoprotein
FETUB_BOVIN	Fetuin-B
FIBA_BOVIN	Fibrinogen alpha chain
FIBB_BOVIN	Fibrinogen beta chain
FIBG_BOVIN	Fibrinogen gamma-B chain
FINC_BOVIN	Fibronectin
G3P_BOVIN	Glyceraldehyde-3-phosphate dehydrogenase

GALK1_BOVIN	Galactokinase
GDIB_BOVIN	Rab GDP dissociation inhibitor beta
GELS_BOVIN	Gelsolin
GLYC_BOVIN	Serine hydroxymethyltransferase, cytosolic
GPC1_BOVIN	Glypican-1
H2A1_BOVIN	Histone H2A type 1
H2A2C_BOVIN	Histone H2A type 2-C
H2B1K_BOVIN	Histone H2B type 1-K
H4_BOVIN	Histone H4
HABP2_BOVIN	Hyaluronan-binding protein 2
HBA_BOVIN	Hemoglobin subunit alpha
HBBF_BOVIN	Hemoglobin fetal subunit beta
HGFL_BOVIN	Hepatocyte growth factor-like protein
HMGB2_BOVIN	High mobility group protein B2
HPLN1_BOVIN	Hyaluronan and proteoglycan link protein 1
HS90A_BOVIN	Heat shock protein HSP 90-alpha
HSP7C_BOVIN	Heat shock cognate 71 kDa protein
HSPB1_BOVIN	Heat shock protein beta-1
IBP5_BOVIN	Insulin-like growth factor-binding protein 5
IPSP_BOVIN	Plasma serine protease inhibitor
ITIH1_BOVIN	Inter-alpha-trypsin inhibitor heavy chain H1
ITIH3_BOVIN	Inter-alpha-trypsin inhibitor heavy chain H3
ITIH4_BOVIN	Inter-alpha-trypsin inhibitor heavy chain H4
K1C10_BOVIN	Keratin, type I cytoskeletal 10
KNG1_BOVIN	Kininogen-1
LASP1_BOVIN	LIM and SH3 domain protein 1
LDHA_BOVIN	L-lactate dehydrogenase A chain
LECT2_BOVIN	Leukocyte cell-derived chemotaxin-2
MIF_BOVIN	Macrophage migration inhibitory factor
MYH10_BOVIN	Myosin-10
MYL6_BOVIN	Myosin light polypeptide 6
NDKB_BOVIN	Nucleoside diphosphate kinase B
NP1L1_BOVIN	Nucleosome assembly protein 1-like 1
NRX1A_BOVIN	Neurexin-1
OLM2B_BOVIN	Olfactomedin-like protein 2B
PDIA1_BOVIN	Protein disulfide-isomerase
PEDF_BOVIN	Pigment epithelium-derived factor
PERI_BOVIN	Peripherin
PGCA_BOVIN	Aggrecan core protein
PGRP1_BOVIN	Peptidoglycan recognition protein 1

PGS1_BOVIN	Biglycan
PGS2_BOVIN	Decorin
PLCB1_BOVIN	1-phosphatidylinositol 4,5-bisphosphate phosphodiesterase beta-1
PLMN_BOVIN	Plasminogen
PRDX1_BOVIN	Peroxiredoxin-1
PSB6_BOVIN	Proteasome subunit beta type-6
PSMD2_BOVIN	26S proteasome non-ATPase regulatory subunit 2
RGN_BOVIN	Regucalcin
RL12_BOVIN	60S ribosomal protein L12
RL7_BOVIN	60S ribosomal protein L7
RLA0_BOVIN	60S acidic ribosomal protein P0
ROA1_BOVIN	Heterogeneous nuclear ribonucleoprotein A1
RS28_BOVIN	40S ribosomal protein S28
RSSA_BOVIN	40S ribosomal protein SA
SAHH_BOVIN	Adenosylhomocysteinase
SDC1_BOVIN	Syndecan-1
SDC2_BOVIN	Syndecan-2
SPON1_BOVIN	Spondin-1
SPP24_BOVIN	Secreted phosphoprotein 24
TAGL2_BOVIN	Transgelin-2
TBA1B_BOVIN	Tubulin alpha-1B chain
TBB2B_BOVIN	Tubulin beta-2B chain
TBB4A_BOVIN	Tubulin beta-4A chain
TCO2_BOVIN	Transcobalamin-2
TERA_BOVIN	Transitional endoplasmic reticulum ATPase
THRB_BOVIN	Prothrombin
TPM1_BOVIN	Tropomyosin alpha-1 chain
TRY1_BOVIN	Cationic trypsin
TSP1_BOVIN	Thrombospondin-1
TSP4_BOVIN	Thrombospondin-4
UBA1_BOVIN	Ubiquitin-like modifier-activating enzyme 1
VIME_BOVIN	Vimentin
YBOX1_BOVIN	Nuclease-sensitive element-binding protein 1

Table S9: List of shared proteins recovered from AgNPs after 24 h incubation with NIH-3T3 medium

Abbreviation	Name
A1AT_BOVIN	Alpha-1-antiproteinase
A2MG_BOVIN	Alpha-2-macroglobulin
ACTB_BOVIN	Actin, cytoplasmic 1
ALBU_BOVIN	Serum albumin

ANXA2_BOVIN	Annexin A2
APOA1_BOVIN	Apolipoprotein A-I
APOE_BOVIN	Apolipoprotein E
BIP_BOVIN	Endoplasmic reticulum chaperone BiP
CFAB_BOVIN	Complement factor B
CFAH_BOVIN	Complement factor H
CH10_BOVIN	10 kDa heat shock protein, mitochondrial
CH60_BOVIN	60 kDa heat shock protein, mitochondrial
CO1A1_BOVIN	Collagen alpha-1(I) chain
CO3_BOVIN	Complement C3
CO4_BOVIN	Complement C4 (Fragments)
EF1A1_BOVIN	Elongation factor 1-alpha 1
ENOA_BOVIN	Alpha-enolase
ENPL_BOVIN	Endoplasmin
EZRI_BOVIN	Ezrin
FIBA_BOVIN	Fibrinogen alpha chain
FINC_BOVIN	Fibronectin
GELS_BOVIN	Gelsolin
H2A1_BOVIN	Histone H2A type 1
H2A2C_BOVIN	Histone H2A type 2-C
H2B1K_BOVIN	Histone H2B type 1-K
H4_BOVIN	Histone H4
HBA_BOVIN	Hemoglobin subunit alpha
HBBF_BOVIN	Hemoglobin fetal subunit beta
HS90A_BOVIN	Heat shock protein HSP 90-alpha
HSP7C_BOVIN	Heat shock cognate 71 kDa protein
ITIH3_BOVIN	Inter-alpha-trypsin inhibitor heavy chain H3
ITIH4_BOVIN	Inter-alpha-trypsin inhibitor heavy chain H4
KNG1_BOVIN	Kininogen-1
LASP1_BOVIN	LIM and SH3 domain protein 1
LDHB_BOVIN	L-lactate dehydrogenase B chain
MYL9_BOVIN	Myosin regulatory light polypeptide 9
PLMN_BOVIN	Plasminogen
PRDX1_BOVIN	Peroxiredoxin-1
RL14_BOVIN	60S ribosomal protein L14
RS28_BOVIN	40S ribosomal protein S28
TBA1B_BOVIN	Tubulin alpha-1B chain
TBB2B_BOVIN	Tubulin beta-2B chain
THRB_BOVIN	Prothrombin
TRY1_BOVIN	Cationic trypsin

TSP1_BOVIN	Thrombospondin-1
VIME_BOVIN	Vimentin

Table S10: List of shared proteins recovered from AgNPs after 24 h incubation with MCF7/Caco2 medium

Abbreviation	Name
A1AT_BOVIN	Alpha-1-antiproteinase
A1BG_BOVIN	Alpha-1B-glycoprotein
A2MG_BOVIN	Alpha-2-macroglobulin
ACTB_BOVIN	Actin, cytoplasmic 1
ALBU_BOVIN	Serum albumin
APOA1_BOVIN	Apolipoprotein A-I
APOE_BOVIN	Apolipoprotein E
ARF1_BOVIN	ADP-ribosylation factor 1
BIP_BOVIN	Endoplasmic reticulum chaperone BiP
CFAB_BOVIN	Complement factor B
CFAH_BOVIN	Complement factor H
CLUS_BOVIN	Clusterin
CO1A1_BOVIN	Collagen alpha-1(I) chain
CO3_BOVIN	Complement C3
CO4_BOVIN	Complement C4 (Fragments)
CO9_BOVIN	Complement component C9
EF1A1_BOVIN	Elongation factor 1-alpha 1
ENOA_BOVIN	Alpha-enolase
ENPL_BOVIN	Endoplasmin
F13A_BOVIN	Coagulation factor XIII A chain (Fragment)
FA5_BOVIN	Coagulation factor V
FETA_BOVIN	Alpha-fetoprotein
FETUA_BOVIN	Alpha-2-HS-glycoprotein
FETUB_BOVIN	Fetuin-B
FIBA_BOVIN	Fibrinogen alpha chain
FIBB_BOVIN	Fibrinogen beta chain
FIBG_BOVIN	Fibrinogen gamma-B chain
FINC_BOVIN	Fibronectin
GELS_BOVIN	Gelsolin
H2A1_BOVIN	Histone H2A type 1
H2B1K_BOVIN	Histone H2B type 1-K
H4_BOVIN	Histone H4
HBA_BOVIN	Hemoglobin subunit alpha

HBBF_BOVIN	Hemoglobin fetal subunit beta
HS90A_BOVIN	Heat shock protein HSP 90-alpha
HSP7C_BOVIN	Heat shock cognate 71 kDa protein
ITIH1_BOVIN	Inter-alpha-trypsin inhibitor heavy chain H1
ITIH3_BOVIN	Inter-alpha-trypsin inhibitor heavy chain H3
ITIH4_BOVIN	Inter-alpha-trypsin inhibitor heavy chain H4
KNG1_BOVIN	Kininogen-1
MIF_BOVIN	Macrophage migration inhibitory factor
MYH10_BOVIN	Myosin-10
MYL6_BOVIN	Myosin light polypeptide 6
PERI_BOVIN	Peripherin
PGRP1_BOVIN	Peptidoglycan recognition protein 1
PLMN_BOVIN	Plasminogen
PRDX1_BOVIN	Peroxiredoxin-1
RGN_BOVIN	Regucalcin
SPP24_BOVIN	Secreted phosphoprotein 24
TAGL2_BOVIN	Transgelin-2
TBA1B_BOVIN	Tubulin alpha-1B chain
THRB_BOVIN	Prothrombin
TSP1_BOVIN	Thrombospondin-1
TSP4_BOVIN	Thrombospondin-4

Table S11: List of proteins shared by the protein corona, formed after 24 hours of incubation with MCF7/Caco2 medium, of negatively charged NPs (AgNPs-cit and AgNPs-PSS).

Abbreviation	Name
ABHEB_BOVIN	Protein ABHD14B
CATA_BOVIN	Catalase
CATC_BOVIN	Dipeptidyl peptidase 1
CBPB2_BOVIN	Carboxypeptidase B2
CFAD_BOVIN	Complement factor D
CL43_BOVIN	Collectin-43
CO7_BOVIN	Complement component C7
COMP_BOVIN	Cartilage oligomeric matrix protein
CPNS1_BOVIN	Calpain small subunit 1
CTHL1_BOVIN	Cathelicidin-1
CTHL4_BOVIN	Cathelicidin-4
DHSO_BOVIN	Sorbitol dehydrogenase
EF1G_BOVIN	Elongation factor 1-gamma

F16P1_BOVIN	Fructose-1,6-bisphosphatase 1
FA12_BOVIN	Coagulation factor XII
FKB1A_BOVIN	Peptidyl-prolyl cis-trans isomerase FKBP1A
GLO2_BOVIN	Hydroxyacylglutathione hydrolase, mitochondrial
HEBP1_BOVIN	Heme-binding protein 1
HEM2_BOVIN	Delta-aminolevulinic acid dehydratase
HP20_BOVIN	Protein HP-20 homolog
IBP2_BOVIN	Insulin-like growth factor-binding protein 2
IBP6_BOVIN	Insulin-like growth factor-binding protein 6
K2C79_BOVIN	Keratin, type II cytoskeletal 79
KCRB_BOVIN	Creatine kinase B-type
MDHC_BOVIN	Malate dehydrogenase, cytoplasmic
METK1_BOVIN	S-adenosylmethionine synthase isoform type-1
MOES_BOVIN	Moesin
PDLI1_BOVIN	PDZ and LIM domain protein 1
PEBP1_BOVIN	Phosphatidylethanolamine-binding protein 1
PPIA_BOVIN	Peptidyl-prolyl cis-trans isomerase A
PRDX2_BOVIN	Peroxiredoxin-2
PRDX6_BOVIN	Peroxiredoxin-6
PSA6_BOVIN	Proteasome subunit alpha type-6
PSA7_BOVIN	Proteasome subunit alpha type-7
PSB4_BOVIN	Proteasome subunit beta type-4
SEPT2_BOVIN	Septin-2
SERPH_BOVIN	Serpin H1
TAGL_BOVIN	Transgelin
TBA4A_BOVIN	Tubulin alpha-4A chain
TES_BOVIN	Testin
TKT_BOVIN	Transketolase
TPIS_BOVIN	Triosephosphate isomerase
TPM2_BOVIN	Tropomyosin beta chain
TTHY_BOVIN	Transthyretin
VASP_BOVIN	Vasodilator-stimulated phosphoprotein

Table S12: List of proteins shared by the protein corona, formed after 24 hours of incubation with MCF7/Caco2 medium, of positively charged NPs (AgNPs-PAH and AgNPs-PDDA).

Abbreviation	Name
ANXA2_BOVIN	Annexin A2
ATPB_BOVIN	ATP synthase subunit beta, mitochondrial
CH60_BOVIN	60 kDa heat shock protein, mitochondrial
CO3A1_BOVIN	Collagen alpha-1(III) chain
GPC1_BOVIN	Glypican-1
NRX1A_BOVIN	Neurexin-1
OLM2B_BOVIN	Olfactomedin-like protein 2B
PGCA_BOVIN	Aggrecan core protein
PGS1_BOVIN	Biglycan
PGS2_BOVIN	Decorin
RLA0_BOVIN	60S acidic ribosomal protein P0
RSSA_BOVIN	40S ribosomal protein SA
TPM1_BOVIN	Tropomyosin alpha-1 chain
UBA1_BOVIN	Ubiquitin-like modifier-activating enzyme 1
